# Combining bioinformatics, cheminformatics, functional genomics and whole organism approaches for identifying epigenetic drug targets in *Schistosoma mansoni*

**DOI:** 10.1016/j.ijpddr.2018.10.005

**Published:** 2018-11-13

**Authors:** Gilda Padalino, Salvatore Ferla, Andrea Brancale, Iain W. Chalmers, Karl F. Hoffmann

**Affiliations:** a The Institute of Biological, Environmental and Rural Sciences (IBERS), Aberystwyth University, SY23 3DA, Wales, UK; b School of Pharmacy and Pharmaceutical Sciences, Cardiff University, Cardiff, CF10 3NB, United Kingdom

**Keywords:** Anthelmintic drug discovery, Neglected tropical diseases, *Schistosoma mansoni*, Epigenetics, Lysine specific demethylase

## Abstract

Schistosomiasis endangers the lives of greater than 200 million people every year and is predominantly controlled by a single class chemotherapy, praziquantel (PZQ). Development of PZQ replacement (to combat the threat of PZQ insensitivity/resistance arising) or combinatorial (to facilitate the killing of PZQ-insensitive juvenile schistosomes) chemotherapies would help sustain this control strategy into the future. Here, we re-categorise two families of druggable epigenetic targets in *Schistosoma mansoni*, the histone methyltransferases (HMTs) and the histone demethylases (HDMs). Amongst these, a *S. mansoni* Lysine Specific Demethylase 1 (SmLSD1, Smp_150560) homolog was selected for further analyses. Homology modelling of SmLSD1 and *in silico* docking of greater than four thousand putative inhibitors identified seven (L1 – L7) showing more favourable binding to the target pocket of SmLSD1 vs *Homo sapiens* HsLSD1; six of these seven (L1 – L6) plus three structural analogues of L7 (L8 – L10) were subsequently screened against schistosomula using the Roboworm anthelmintic discovery platform. The most active compounds (L10 - pirarubicin > L8 – danunorubicin hydrochloride) were subsequently tested against juvenile (3 wk old) and mature (7 wk old) schistosome stages and found to impede motility, suppress egg production and affect tegumental surfaces. When compared to a surrogate human cell line (HepG2), a moderate window of selectivity was observed for the most active compound L10 (selectivity indices - 11 for schistosomula, 9 for juveniles, 1.5 for adults). Finally, RNA interference of SmLSD1 recapitulated the egg-laying defect of schistosomes co-cultivated in the presence of L10 and L8. These preliminary results suggest that SmLSD1 represents an attractive new target for schistosomiasis; identification of more potent and selective SmLSD1 compounds, however, is essential. Nevertheless, the approaches described herein highlight an interdisciplinary strategy for selecting and screening novel/repositioned anti-schistosomals, which can be applied to any druggable (epigenetic) target encoded by the parasite's genome.

## Highlights

• *Schistosoma mansoni* contains 27 histone methyltransferases (HMTs) and 14 histone demethylases (HDMs).
• *S. mansoni* lysine specific demethylase 1 (SmLSD1) is a druggable target.
• Schistosomes treated with the putative SmLSD1 inhibitor pirarubicin or siRNAs targeting SmLSD1 are less fecund.

## Graphical abstract

**Figure:**
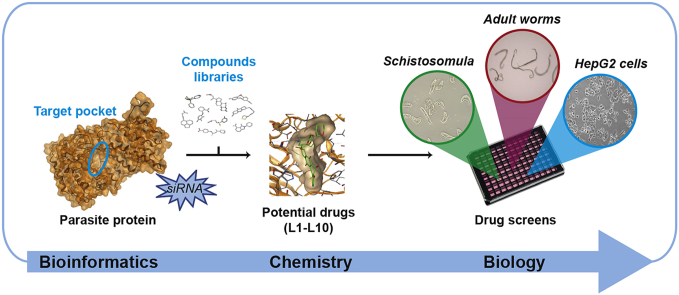

## 1 Introduction

Until a safe, reliable and efficacious prophylactic vaccine is developed, human schistosomiasis will continue to be primarily treated by anthelmintic chemotherapy. Currently, the drug of choice used in schistosomiasis control throughout endemic or emerging areas is praziquantel, a pyrazino-isoquinolone compound developed over 40 years ago as part of Merck's effort in developing a new tranquilizer class (reviewed in (Cioli and Pica-Mattoccia, 2003)). While praziquantel is safe, effective, relatively inexpensive to produce and operationally easy to deliver (Cioli et al., 2014), its inability to kill immature parasites (Wu et al., 2011) and its unclear mechanism of action (possibly as a G-protein coupled receptor antagonist (Chan et al., 2017)) raises concerns over its sustainability in helping to meet the World Health Organisation's objective of regional schistosomiasis elimination by 2020 and beyond (WHO, 2012). Added to these concerns is the spectre of praziquantel-insensitive or resistant parasites developing (Cupit and Cunningham, 2015). Therefore, new praziquantel replacement (if resistance develops) or combinatorial (with activity against immature parasites) anti-schistosomals are urgently needed for combatting a disease that currently affects greater than 200 million people annually (Colley et al., 2014).

Over the past decade, we, and others, have been involved in developing and applying high-throughput methods for selecting targets or compounds integral to early stage schistosome drug discovery projects (Kuntz et al., 2007; Caffrey et al., 2009; Fitzpatrick et al., 2009b; Peak et al., 2010; Rojo-Arreola et al., 2014; Mansour et al., 2016; Aguiar et al., 2017). Our specific efforts have identified (amongst others) arginase as a suitable schistosome drug target (Fitzpatrick et al., 2009a; Hai et al., 2014) and diterpenoids as potential, broad-acting anthelmintic compounds (Edwards et al., 2015; Crusco et al., 2018). Due to a growing realisation that schistosome development is regulated by epigenetic processes (Geyer and Hoffmann, 2012), we have now specifically focused on schistosome epigenetic targets as an additional repository for progressing anti-schistosomal efforts (Geyer et al., 2011, 2018a, 2018b; Cosseau et al., 2016; Roquis et al., 2018). Other investigators have also interrogated epigenetic space (i.e. the processes responsible for inheritable phenotypes that do not involve genome alterations) for schistosome drug discovery with most attention directed towards the identification and inhibition of histone modifying enzymes (HMEs) (e.g. (Azzi et al., 2009; Pierce et al., 2011; Pierce et al., 2012; Marek et al., 2013; Cabezas-Cruz et al., 2014; Carneiro et al., 2014). As HMEs are critical regulators of developmental fate and disease pathogenesis in other metazoans (Butler et al., 2012), their selection as druggable epigenetic targets in early-stage schistosome drug discovery projects is well justified.

In this study, we reclassified two functionally opposing HME families in *Schistosoma mansoni*, the histone methyltransferases (HMTs) and histone demethylases (HDMs). Amongst these epigenetic enzymes, a *S. mansoni* lysine specific demethylase 1 (SmLSD1, Smp_150560) homolog was selected for an early stage drug discovery investigation based on the importance and role of *Homo sapiens* lysine specific demethylase 1 (HsLSD1) in essential biological processes (Maiques-Diaz and Somervaille, 2016; Ismail et al., 2018). Using SmLSD1 as a model, we illustrate how bioinformatics, cheminformatics, functional genomics and robotic whole organism screening can be synergistically applied to any druggable (epigenetic) target in the *S. mansoni* genome.

## 2 Materials and methods

### 2.1 Ethics statement

All procedures performed on mice (project license PPL 40/3700) adhered to the United Kingdom Home Office Animals (Scientific Procedures) Act of 1986 as well as the European Union Animals Directive 2010/63/EU and were approved by Aberystwyth University's (AU) Animal Welfare and Ethical Review Body (AWERB).

### 2.2 Parasite material

A Puerto Rican strain (NMRI) of *Schistosoma mansoni* was used throughout the study and passaged between *Mus musculus* (Tuck Ordinary; TO) and *Biomphalaria glabrata* (NMRI albino and pigmented hybrid (Geyer et al., 2017)) hosts. Cercariae were shed from both *B. glabrata* strains by exposure to light in an artificially heated room (26 °C) for 1 h and used to percutaneously infect *M. musculus* (180 cercariae/mouse for the generation of 7 wk old adult worms or 4000 cercariae/mouse for the generation of 3 wk old juvenile worms) (Smithers and Terry, 1965). Cercariae were also used to generate mechanically transformed schistosomula (Colley and Wikel, 1974). Adult (7 wk post infection) and juvenile (3 wk post infection) schistosomes were obtained from *M. musculus* by portal vein perfusion (Duvall and Dewitt, 1967). Schistosomula, juvenile and adult worms were used for compound screening. Adult worms were also used for RNA interference (RNAi).

### 2.3 Identification of histone methyltransferases (HMTs) and histone demethylases (HDMs)

The identification of *S. mansoni* HMTs and HDMs was performed using two methodologies. The first methodology focused on sequence similarity between the HMTs/HDMs from *H. sapiens* and *S. mansoni*. Here, representative examples of *H. sapiens* HMTs/HDMs were downloaded from Uniprot (UniProt, 2009) and their protein sequences used as queries for protein BLAST (BLASTp) searches against the predicted protein database derived from the *S. mansoni* genome (v 7.0) hosted in NCBI (https://blast.ncbi.nlm.nih.gov/Blast.cgi) and Wormbase-Parasite (Howe et al., 2017) using default settings. The human HMTs/HDMs sequences (and their Uniprot access numbers) used were as follows: Histone-lysine N-methyltransferase EZH2 (Q15910), Histone-lysine N-methyltransferase 2A (known as MLL1, Q03164), Histone-lysine N-methyltransferase SETD2 (Q9BYW2), Histone-lysine N-methyltransferase SUV39H2 (Q9H5I1), N-lysine methyltransferase SMYD2 (Q9NRG4), Histone-lysine N-methyltransferase, H3 lysine-79 specific (known as DOT1L, Q8TEK3), Protein arginine N-methyltransferase 1 (Q63009), Histone-arginine methyltransferase CARM1 (Q86X55), Lysine-specific histone demethylase 1A (O60341), Lysine-specific demethylase 4A (known as Jmjd2, O75164), JmjC domain-containing protein 5 (Q8N371) and Bifunctional arginine demethylase and lysyl-hydroxylase JMJD6 (Q6NYC1). For the BLASTp searches, full-length and catalytic domain peptide sequences were used as queries against the *S. mansoni* genome (v. 7.0). *S. mansoni* sequences were recognized as HMTs or HDMs only if they demonstrated high sequence similarity (an *E* value of 1e10^−5^ or lower) to human HMTs/HDMs and were observed (by visual inspection of the BLASTp pairwise alignments) to contain conservation over the catalytic domain as defined by InterPro (Finn et al., 2017), Pfam (Punta et al., 2012), PROSITE (Hofmann et al., 1999) or SMART (Schultz et al., 1998).

To improve the robustness of our searches, a second methodology was applied based on the retention of conserved catalytic domain residues responsible for HMT and HDM enzymatic activity. Here, HMT and HDM domain identifiers were selected within Interpro, Pfam, PROSITE and SMART (see Supplementary Table 1). Each signature was used as the query in Wormbase-Parasite BioMart to identify all putative *S. mansoni* proteins containing the aforementioned catalytic domains.

The combination of these approaches led to the identification of 27 HMTs (three novel members) and 14 HDMs (three novel members) in *S. mansoni*. The HMTs were grouped into 20 Protein Lysine Methyl Transferases (PKMTs), 1 PR domain containing methyltransferase (PRDM), 1 DOT1 Like Histone Lysine Methyltransferase (DOT1L) and 5 Protein Arginine Methyl Transferases (PRMTs). The HDMs were grouped into 3 Lysine Specific Demethylases (LSDs) and 11 Jumonji domain-containing proteins (JMJDs). The most closely related *Schistosoma haematobium* and *Schistosoma japonicum* homologous sequences for these 27 SmHMTs and 14 SmHDMs were also identified by BLASTp interrogation of WormBase-Parasite. For the BLASTp searches, full-length protein sequences of each SmHMT and SmHDM were used as queries against the *S. haematobium* and *S. japonicum* predicted protein datasets. *S. haematobium* and *S. japonicum* sequences were recognized as the most closely related HMT and HDM homologs respectively (Sh/SjHMTs and Sh/SjHDMs) only if they demonstrated high sequence similarity (an *E* value of 1e10^−50^ or lower). Only the top *S. haematobium* or *S. japonicum* hit (as defined by score) was included (Supplementary Table 2).

### 2.4 Multiple sequence alignment and phylogenetic analyses

Alignments of the catalytic domain amino acid sequences within the identified SmHMTs and SmHDMs were performed with MUSCLE (Multiple Sequence Comparison by Log Expectation) using the default parameters (Edgar, 2004). The multiple sequence alignments were interrogated to determine regions of conservation using GBLOCKs 0.91b set to identify smaller block sizes with less strict flanking positions (Castresana, 2000). For the HMT phylogenetic analysis, four regions covering a total of 50 amino acids were chosen by the GBLOCKS software, whereas the HDM analysis used three regions containing a total of 39 amino acids selected by the GBLOCKs software. Phylogenetic trees were constructed by MEGA7 using the neighbour-joining method based on the JTT matrix-based model with default settings and 1000 bootstrap replications (Kumar et al., 2016) and visualised using the Tree Of Life v1.0 (Ciccarelli et al., 2006).

### 2.5 SmHMT and SmHDM domain classifications

The amino acid positions of *S. mansoni* HMT and HDM domains having InterPro, SMART and Pfam identifiers were extracted from Wormbase-Parasite. These catalytic domains were defined as follows: SET, **S**u(var)3–9, **E**nhancer-of-zeste and **T**rithorax domain for the PKMTs; DOT1, **D**isruptor **o**f the **t**elomeric silencing 1 for DOT1L; PRMT core, **P**rotein Arginine (**R**) **M**ethyl**t**ransferase core domain for PRMTs; AOL, **A**mine **O**xidase-**l**ike domain for LSDs; JMJC, **J**u**m**on**j**i **C** domain and JMJN, **J**u**m**on**j**i **N** domain for the JMJDs. The start and end position of these domains were confirmed, or modified if needed, according to the multiple sequence alignments.

The architecture of these proteins was then populated with other N-terminal and/or C-terminal domains known from the literature to be associated with the aforementioned catalytic domains. Graphical representation of these domains within the schistosome HMTs and HDMs was prepared using Illustrator for Biological Sequences (IBS, (Liu et al., 2015)).

### 2.6 *Smhmt* and *Smhdm* transcription profiles

Data from the 37,632 element *S. mansoni* long-oligonucleotide DNA microarray studies of Fitzpatrick et al. (2009b) were interrogated to find the expression profile of the 27 *Smhmt*s and 14 *Smhdm*s across 14 different lifecycle stages. Here, Log2 normalised gene expression data were subjected to hierarchical agglomerative clustering (Euclidean distance and complete linkage) using the R statistical programming language and the Bioconductor package gplots (Gentleman et al., 2004). The analysed data were then illustrated as a heat map. Raw and normalised fluorescent intensity values are available via Array Express under the experimental accession number E-MEXP-2094. Oligonucleotide probes corresponding to each *Smhmt* and *Smhdm* as well as their corresponding log2 expression values are found in Supplementary Table 2.

### 2.7 Homology modelling

The homology model of Smp_150560 (*S. mansoni* Lysine Specific Demethylase 1, SmLSD1) was prepared with the Molecular Operating Environment (MOE) 2015.10 (Chemical Computing Group, 2018) homology tool using a single template approach. The template selected for SmLSD1 was the three-dimensional structure of *H. sapiens* LSD1 (HsLSD1; Protein Data Bank – PDB - (Berman et al., 2000) identification code 2V1D, chain A) (Forneris et al., 2007). The sequence identity between SmLSD1 and the human template was 41% with a sequence coverage over 60%, hence well within the acceptable range for comparative modelling techniques (Baker and Sali, 2001).

An induced fit option was selected to take into account the presence of the substrate (histone H3) and cofactor (Flavin Adenine Dinucleotide, FAD). Ten different intermediate models were built and minimised using Amber94 before refining the final model, which generates a Cartesian average of the 10 generated intermediates. The final model was confirmed by I-TASSER (Iterative Threading ASSEmbly Refinement) (Yang et al., 2015), Robetta (Kim et al., 2004) and SWISS-MODEL (Waterhouse et al., 2018) with the overall quality of the model evaluated by RAMPAGE Ramachandran Plot analysis (http://mordred.bioc.cam.ac.uk/∼rapper/rampage.php).

### 2.8 Compound docking to SmLSD1

Compounds capable of binding to SmLSD1 were identified using Glide docking software within Maestro v10.1 (Maestro, 2015). To initiate docking in either software suite, selection of compounds and preparation of targets was necessary (Supplementary Fig. 1). Firstly, a library of commercially available compounds was downloaded from Specs (www.specs.net, natural compounds and fragment-based library) in *sdf* format. These compounds (4,532) were then processed by Maestro's LigPrep tool to include all the partial charges, the appropriate protonation state at the defined pH, the proper tautomeric states, the bond lengths and angles. Secondly, the crystal structure of HsLSD1 was downloaded from PDB (PDB code 2V1D) and the three-dimensional structure of SmLSD1 was prepared by homology modelling as described above. The two structures were processed with the Schrödinger Protein Preparation Wizard tool using the OPLS_2005 force field to assign partial charges, bond orders, cap residues, add hydrogen atoms, fill in missing loops as well as side chains and to minimize the protein structure relieving any steric clashes. Thirdly, a 12 Å docking grid (inner-box 10 Å and outer-box 22 Å) was computationally built using, as a centroid, the structure of the histone H3 that was used for the co-crystallisation of HsLSD1 as well as for the induced-fit homology modelling of SmLSD1.

Docking simulations for virtual screening of Specs compounds with SmLSD1 were next performed in Glide using the standard precision function (SP; all 4532 compounds) and the results were refined using the more accurate extra precision (XP) function. In all cases, default parameters were selected and ten output poses (conformations) per input ligand were included in the solution. From the 2500 solutions (conformations) ranked according to the Glide XP scoring function, 500 distinct compounds were identified and retained for HsLSD1 (PDB code 2V1D) docking. The compounds with a more favourable docking score (XP score) for SmLSD1 compared to HsLSD1 were considered hits and the top 100 were selected for further processing.

SmLSD1 docking results (Glide XP) of the 100 selected compounds were saved in a single *mol2* file and the docking poses were visually inspected for their binding mode in MOE. The docking poses of each of the 100 selected compounds were evaluated according to the ability of each compound to occupy the target pocket, the number of interactions between the compound and the target protein, the selection of different chemical scaffolds and the potential chemical instability or toxicity (i.e. presence of a nitro group) of the compounds. From this iterative approach, seven putative SmLSD1 inhibitors (L1-L7) and three structural L7 analogues (L8 - L10) were identified (Supplementary Table 3).

### 2.9 Compounds and compound storage

Six of the seven putative SmLSD1 inhibitors (L1-L6) were obtained from Specs (Supplementary Table 3). As L7 was not commercially available, three structural analogues (L8 - L10) were purchased from Sigma Aldrich (UK) (Supplementary Table 3). All compounds were obtained as powder stocks, solubilised in DMSO to a stock concentration of 10 mM and stored at −20 °C until required. Positive controls for *S. mansoni* screens included praziquantel (Sigma-Aldrich, UK) and/or auranofin (Sigma-Aldrich, UK), which were also diluted in DMSO to a stock concentration of 10 mM and working concentration of 1.6 mM.

### 2.10 Whole organism screening of *S. mansoni* schistosomula, juveniles and adults

Mechanically-transformed schistosomula were screened on the Roboworm platform as previously described (Nur et al., 2017; Crusco et al., 2018). Primary screens of L1 - L6 and L8 - L10 were performed at 10 μM final concentration (0.625% DMSO); hits (L8 and L10) at this concentration were subjected to further titration in a secondary screen (10 μM, 5 μM, 2.5 μM, 1.25 μM and 0.625 μM in 0.625% DMSO). Negative (0.625% DMSO) and positive (10 μM auranofin in 0.625% DMSO) control wells were included in all schistosomula screens. For primary screens, each putative SmLSD1 inhibitor was tested in duplicate. For secondary screens, each concentration of L8 and L10 was tested in duplicate and the titration was repeated three times.

Juvenile worm screens (15–20 worms/well) were performed in a 96 well plate format (flat bottom) containing a final volume of 200  μL of media (DMEM (Gibco, Paisley, UK) supplemented with 10% v/v Hepes (Sigma-Aldrich, Gillingham, UK), 10% v/v Foetal Bovine Serum (Gibco, Paisley, UK), 0.7% v/v 200 mM L-Glutamine (Gibco, Paisley, UK) and 1X v/v penicillin-streptomycin (Fisher Scientific, UK)). To each well, L8 and L10 were added at the following concentrations: 20 μM, 15 μM, 10 μM, 5 μM and 2.5 μM and 1.25 μM (in 1.25% DMSO). Parasites were cultured at 37 **°**C in an atmosphere containing 5% CO_2_ for 72 h at which time worm motility was scored between 0 and 4: 0 = dead, 1 = movement of the suckers only and slight contraction of the body, 2 = movement at the anterior and posterior regions only, 3 = full body movement but sluggish and 4 = normal movement. DMSO (negative control, 1.25% DMSO) and praziquantel (positive control, 10 μM in 1.25% DMSO) were included in these juvenile worm assays.

Adult worm screens were initiated as described previously (Crusco et al., 2018). Briefly, schistosome pairs (3 pairs/well in a 48-well microtiter plates) were co-cultivated with L8 and L10 (two-fold titrations between 50 μM and 6.25 μM in 1.25% DMSO) for 72 h at 37 °C in a humidified atmosphere containing 5% CO_2_. As controls, schistosome pairs co-cultivated in DMSO (1.25%) and praziquantel (10 μM in 1.25% DMSO) were included in these assays. After 72 h, motility was scored according to defined WHO-TDR metrics (Ramirez et al., 2007) and eggs deposited in wells were enumerated.

### 2.11 Preparation of *S. mansoni* worms for scanning electron microscopy (SEM)

Adult schistosomes were cultivated (as described above) in sub lethal concentrations of L10 (12.5 μM) for 72 h. Afterwards, schistosomes were prepared for SEM as previously described (Crusco et al., 2018).

### 2.12 HepG2 culture and MTT assay

Overt cytotoxicity of L8 and L10 was assessed on human HepG2 cells as described previously (Crusco et al., 2018). Briefly, 2 × 10^4^ cells/well were seeded in black walled 96-well microtiter plates (Fisher Scientific, Loughborough, UK) and incubated for 24 h at 37 °C in a humidified atmosphere containing 5% CO_2_. To each well, L8 and L10 was subsequently added to obtain final concentrations (in 1.25% DMSO) of 200 μM, 100 μM, 75 μM, 50 μM, 20 μM, 10 μM and 1 μM; negative (DMSO; 1.25%) and positive (1% v/v Triton X-100) control wells were also included. Following a further incubation for 24 h, the MTT assay was performed as previously described (Crusco et al., 2018; Nur et al., 2017). Dose response curves to calculate CC_50_ values were generated in GraphPad Prism 7.02.

### 2.13 RNA interference (RNAi)

Following the perfusion of 7-week infected mice, adult worms were recovered and RNAi performed as previously described (Geyer et al., 2011). *Smlsd1* and non-specific *luciferase (Luc)* siRNA duplexes were purchased from Sigma (siRNA sequences defined in Supplementary Table 4). Briefly, 5 worm pairs were transferred to 4 mm electroporation cuvettes containing DMEM (5.4 g/L D-Glucose, Sigma) supplemented with 2 mM L-glutamine, 10,000 Units/ml penicillin and 10,000 μg/ml streptomycin. siRNA duplexes (5 μg) were subsequently added and worms were electroporated with a single pulse at 125 V for 20 ms using a ECM-830 Square Wave Porator (BTX). Worms were cultured at 37 °C in DMEM (5.4 g/L D-Glucose, Sigma) supplemented with 10% fetal calf serum, 2 mM L-glutamine, 10,000 Units/ml penicillin and 10,000 μg/ml streptomycin in an atmosphere of 5% CO_2_ with a 70% media exchange performed every 24 h. At 48 h, worms were processed for quantitative reverse transcription PCR (qRT-PCR); numbers of eggs produced at this time point were also quantified. Separate worms were maintained in continuous culture for up to 168 h, after which time, deposited eggs were also quantified.

### 2.14 Quantitative reverse transcription PCR (qRT-PCR)

Following RNAi with si*Smlsd1* and si*Luc*, mixed-sex adult worms were incubated for a total of 48 h before processing them for RNA isolation. Briefly, worms were homogenised using a TissueLyser LT (Qiagen, UK) in TRIzol Reagent (Invitrogen, UK) before isolation of total RNA using the Direct-zol RNA Kit (Epigentek, UK). cDNA was then generated, qRT-PCR performed and data analysed as previously described (Fitzpatrick et al., 2009b). qRT-PCR primers are defined in Supplementary Table 4.

### 2.15 Statistics

All Statistical analyses were conducted using GraphPad Prism 7 software. To determine significant differences amongst more than two population means, a Kruskal-Wallis ANOVA followed by Dunn's multiple comparisons test was used. To determine significant differences amongst two population means, a student's t-test assuming unequal variance was performed.

## 3 Results and discussion

### 3.1 Re-characterisation of *S. mansoni* histone methyltransferases (HMTs) and histone demethylases (HDMs)

Recent studies have suggested that components of the schistosome epigenetic machinery, especially histone modifying enzymes (HMEs), should be considered as viable next-generation anthelmintic targets (Pierce et al., 2012; Cabezas-Cruz et al., 2014). The identification and optimisation of compounds that affect *S. mansoni* histone acetyl transferase- (HATs) or histone deacetylase- (HDAC) activities has been the predominant focus of such investigations to date (Andrews et al., 2012; Lancelot et al., 2013; Marek et al., 2013; Carneiro et al., 2014; Schiedel et al., 2015). However, we and others have pursued the biological characterisation and chemotherapeutic targeting of two other epigenetic families in *S. mansoni*, the histone methyltransferases (HMTs) and the histone demethylases (HDMs) (Mansure et al., 2005; Cosseau et al., 2016; Anderson et al., 2017; Roquis et al., 2018). The justification for this approach originates from current clinical interest in developing inhibitors of the homologous human enzymes for therapeutic use in treating cancer and other diseases (McAllister et al., 2016; Zahnow et al., 2016; Hauser et al., 2018). Therefore, identification of selective schistosome HMT and HDM inhibitors could lead to the development of therapeutic strategies for disrupting parasite processes (e.g. reproduction, egg laying) responsible for schistosomiasis pathology and transmission.

**Fig. 1:**
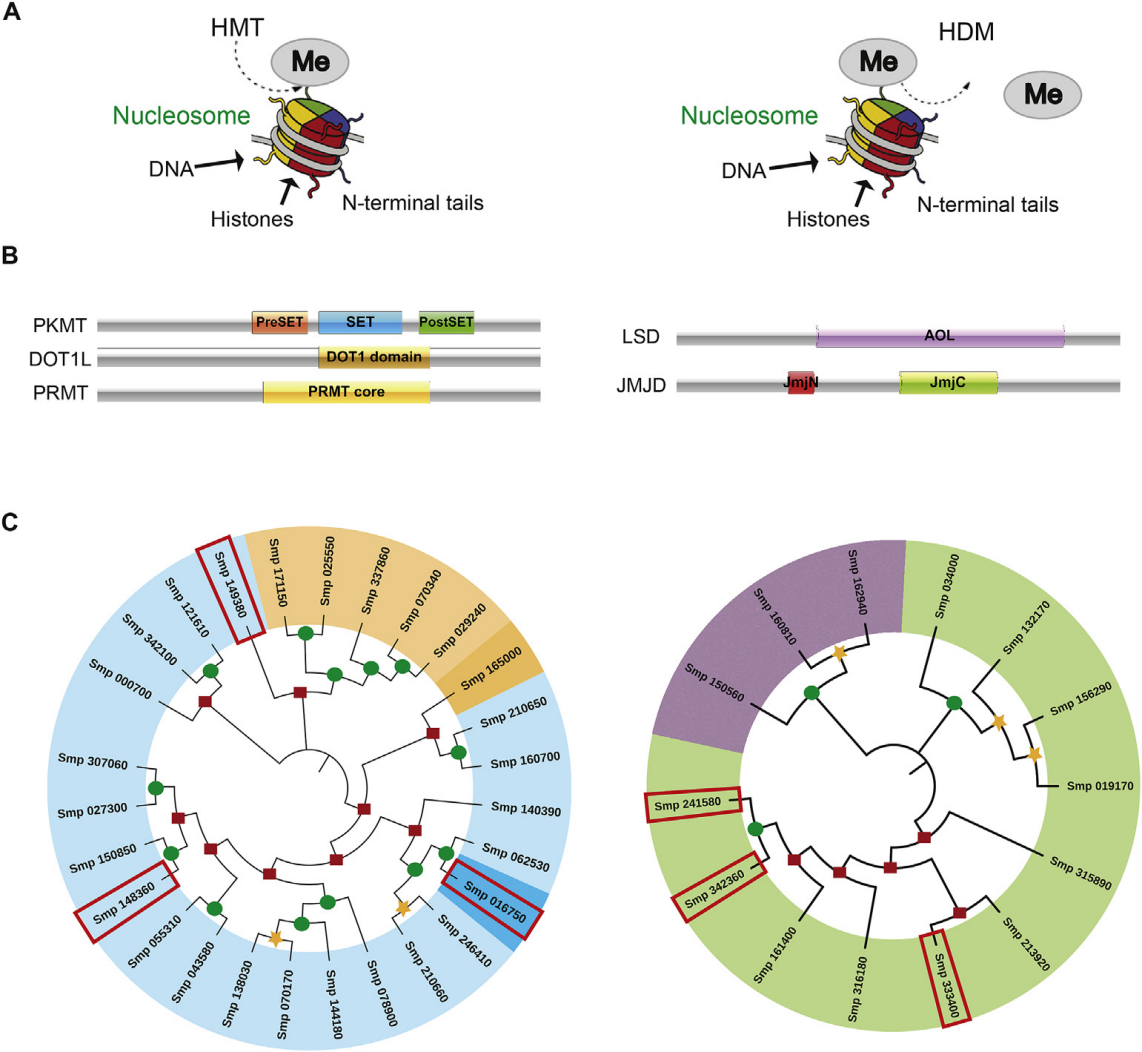
**Re-description of *Schistosoma mansoni* histone methyltransferases (HMTs) and histone demethylases (HDMs).(A)** Schematic mode of action representations for HMT and HDM epigenetic enzymes on nucleosome histones. HMTs add a methyl group (Me) onto the N-terminal tails of histone proteins, whereas HDMs remove this chemical modification. **(B)** Characteristic domains found within the HMT (PKMT, DOT1L, PRMT) and HDM (LSD, JMJD) families. PKMT - Protein Lysine Methyltransferase, DOT1L - Disruptor of telomeric silencing 1-like, PRMT – Protein Arginine Methyltransferase, LSD - Lysine Specific Demethylase, JMJD - Jumonji domain-containing protein, SET - **S**u(var)3–9, **E**nhancer-of-zeste and **T**rithorax, AOL – amine oxidase-like, JmjN – Jumonji N terminal, JmjC – Jumonji C terminal. **(C)** Phylogenetic tree of *S. mansoni* HMTs and HDMs inferred from Neighbour-joining analysis using MEGA as described in the Materials and Methods. The analysis involved 27 HMT and 14 HDM Smps (***S****chistosoma****m****ansoni***p**roteins). Bootstrap support values on the nodes range from 0 to 25 (highlighted with ), between 26 and 74 (highlighted with ) and between 75 and 100 (highlighted with ). New HMTs and HDMs are highlighted in red boxes. A colour code was used for each family of histone modifying enzymes: light blue for PKMT - Protein Lysine Methyltransferases, dark blue for PRDM – SET domain containing PR methyltransferase motifs, dark orange for DOT1L - Disruptor of telomeric silencing 1-like, light orange for PRMT – Protein Arginine Methyltransferases, dark blue for PRDM – PR Domain containing Methyltransferase, purple for LSD - Lysine Specific Demethylases, green for JMJD - Jumonji domain-containing proteins. (For interpretation of the references to colour in this figure legend, the reader is referred to the Web version of this article.)

The initial step in this process was to first identify all putative *S. mansoni* HMT and HDM family members (Fig. 1). As previously reviewed (Cheng et al., 2005; Pedersen and Helin, 2010), HMTs are responsible for adding a methyl group to N-terminal tails of histone (and other) proteins whereas HDMs remove this post-translational modification (Fig. 1A). Within the HMT family are two subfamilies; the Protein Lysine Methyltransferases (PKMTs), which catalyse the methylation of lysine residues and the Protein Arginine Methyltransferases (PRMTs), which enzymatically modify arginine residues. With the exception of DOT1 (**D**isruptor **o**f **t**elomeric silencing, (Singer et al., 1998)) and PRDM (**P**RDI-BF1 and **R**IZ homology **d**omai**n**, (Fog et al., 2012)) containing HMTs, the catalytic region of all other PKMTs contain a well-conserved SET (**S**u(var)3–9, **E**nhancer-of-zeste and **T**rithorax) domain (Fig. 1B). In contrast, the enzymatically active region of all PRMTs is designated as the PRMT core (Fig. 1B).

The use of the SET domain signature (defined by Interpro, Pfam, PROSITE and SMART databases, Supplementary Table 1) led to the identification of 20 putative *S. mansoni* PKMTs in Wormbase parasite (Fig. 1C, light blue Smps). Reassuringly, these results confirmed previous analyses of *S. mansoni* SET-domain containing PKMTs (Cabezas-Cruz et al., 2014) but also identified two new putative members (Smp_149380 and Smp_148360, highlighted in red boxes, Fig. 1C). The identification of these 20 *S. mansoni* PKMTs was confirmed by a second approach based on sequence similarities to human SET-domain containing PKMTs. Here, using human PKMT sequences (full length and catalytic domains) to query the *S. mansoni* genome, only these 20 SmPKMTs were found (*E* value ≤ 1e10^−5^).

Our sequence similarity searches also identified a PRDM-containing PKMT (Smp_016750, Fig. 1C, dark blue Smp outlined in red box). The PRDM family is related to SET domain-containing PKMTs, but with some noticeable differences (reviewed in (Hohenauer and Moore, 2012)). While the catalytic domain of this protein family has 20–30% sequence similarity to the SET domain, PRDM members also contain a variety of different amino acid motifs that differentiate them from other PKMTs (Hohenauer and Moore, 2012). Previously, it was suggested that *S. mansoni* contains 6 PRDMs (Vervoort et al., 2016). However, only one of these proteins (Smp_016750, red box surrounding dark blue ID in Fig. 1C) fitted the criteria used in this study (a divergent SET domain along with other motifs including F/Y/IGP/V and ExNL) (Hohenauer and Moore, 2012). Whether this protein is a catalytically active PKMT is currently unknown.

In addition to the 20 SET-domain and one PRDM-domain containing PKMTs, the complementary approaches used in this study confirmed the presence of only one DOT1L (Smp_165000, dark orange) PKMT and 5 PRMTs (Smp_070340, 029240, 025550, 171150 and 337860, light orange) (Fig. 1C) (Cabezas-Cruz et al., 2014). A detailed architectural analysis of each HMT family member was conducted and demonstrated a rich collection of functional domains important for translating histone methylation into functional cellular phenotypes (Supplementary Fig. 2).

Similar to HMTs, HDMs are usually grouped into two distinct subfamilies based on their proposed mechanisms of action: the Lysine Specific Demethylases (LSDs), which are FAD-dependent amine oxidases and the Jumonji C domain-containing demethylases (JMJDs), which are Fe(II) and 2-oxoglutarate-dependent enzymes. For identification purposes, the catalytic domain signatures of these two subfamilies are the Amine Oxidase-Like (AOL) domain (LSDs) and the Jumonji C terminal domain (JmjC, usually associated with another domain known as Jumonji N terminal - JmjN) (Fig. 1B and Supplementary Table 1). In the case of schistosome LSDs, BLASTp searches in NCBI using the full protein sequence of the human Lysine-specific histone demethylase 1A (O60341) and Lysine-specific histone demethylase 1B (Q8NB78) led to the confirmation of Smp_150560 (*E* value = 2e^−155^) as a putative SmLSD1 and Smp_160810 (E value = 1e^−06^) and Smp_162940 (E value = 1e^−08^) as putative SmLSD2s (Fig. 1B, purple Smps). In support of these findings, the domain architecture of these three Smps were further analysed for additional discriminatory motifs that are characteristic of LSD1 and LSD2 family members (Supplementary Fig. 2). Here, a SWIRM (**Swi**3p, **R**sc8p, and **M**oira) domain was found in all three Smps, but a TOWER domain was only present in Smp_150560. In addition, both Smp_160810 and Smp_162940 contained two zinc finger (zf-C4H2C2 and zf-CW) domains. Based on these features (Ismail et al., 2018), Smp_150560 appears to be the only LSD1 present in the *S. mansoni* genome.

Regarding the JMJDs, the use of JmjC signatures (Supplementary Table 1) led to the identification of 11 putative *S. mansoni* members; three of these also contain the JmjN domain (Fig. 1C, green Smps and Supplementary Fig. 2). The most interesting result was the identification of three previously unknown JMJDs (Smp_342360, Smp_241580 and Smp_333400; highlighted within red boxes in Fig. 1C and Supplementary Fig. 2). The domain architecture of these SmHDMs was analysed in detail (Supplementary Fig. 2) and, similar to the SmHMTs, illustrated the diversity of motifs used for regulating histone (and other protein) methylation states.

Using these bioinformatics-led approaches, the putative schistosome HMTs/HDMs previously identified (Pierce et al., 2012; Cosseau et al., 2016) were confirmed and an additional six new HMTs/HDMs were identified. Based on these analyses, the *S. mansoni* histone methylation machinery is comprised of 27 HMTs and 14 HDMs. Homologous *S. haematobium* and *S. japonicum* HMTs and HDMs (ShHMT/SjHMT or ShHDM/SjHDM), when found, were also recorded (Supplementary Fig. 2).

### 3.2 Longitudinal transcriptional analysis of *S. mansoni* HMTs and HDMs

**Fig. 2:**
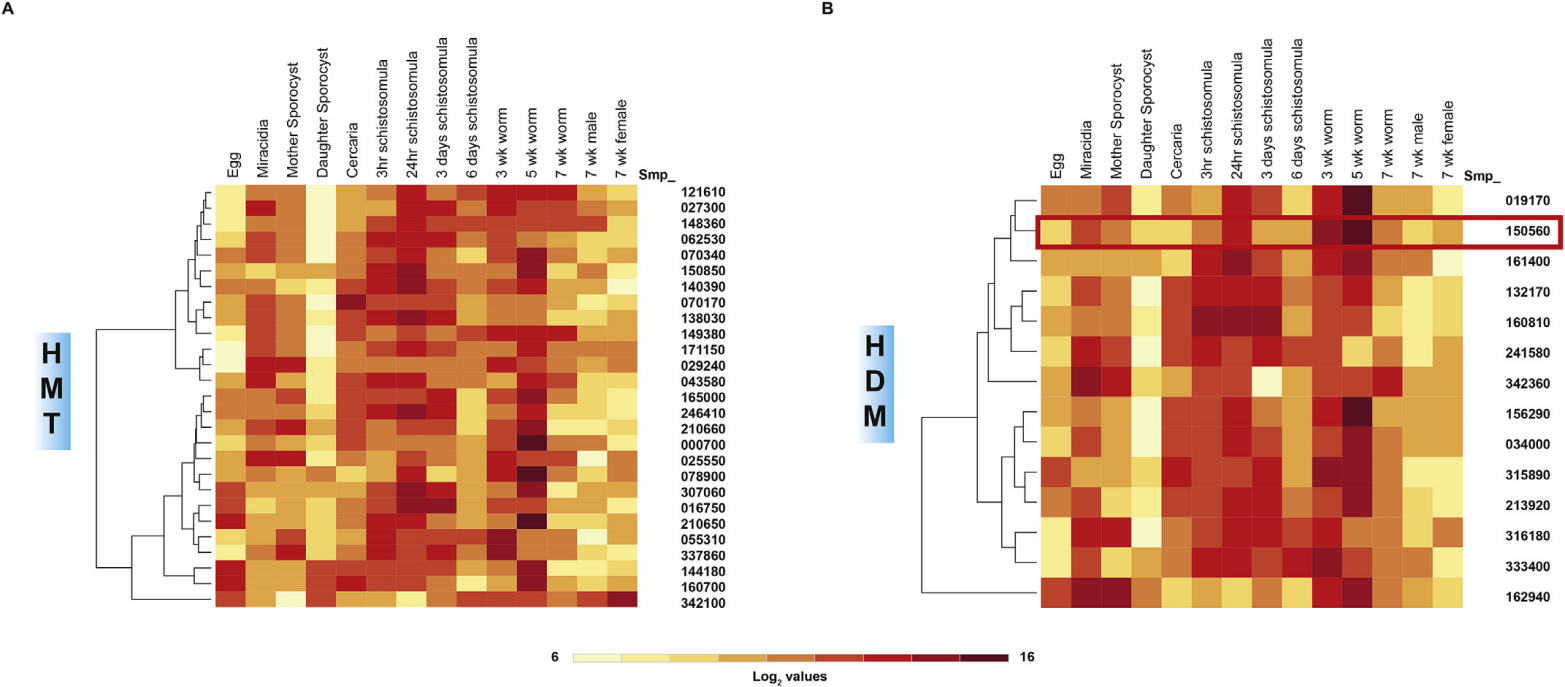
***S. mansoni hmt*s and *hdm*s are differentially expressed throughout development.** DNA microarray analysis and heat map representation of **(A)***hmt* and **(B)***hdm* transcript abundance across 14 schistosome lifecycle stages. The expression level for each *hmt* or *hdm* was calculated as the ratio of individual log2 expression values (obtained from (Fitzpatrick et al., 2009b)) to the median *hmt* or *hdm* log2 expression value (red = up-regulated; yellow = down-regulated). Hierarchical clustering (Euclidean distance and complete linkage) of HMT and HDM Smp IDs (rows) are indicated on both panels. (For interpretation of the references to colour in this figure legend, the reader is referred to the Web version of this article.)

**Fig. 3:**
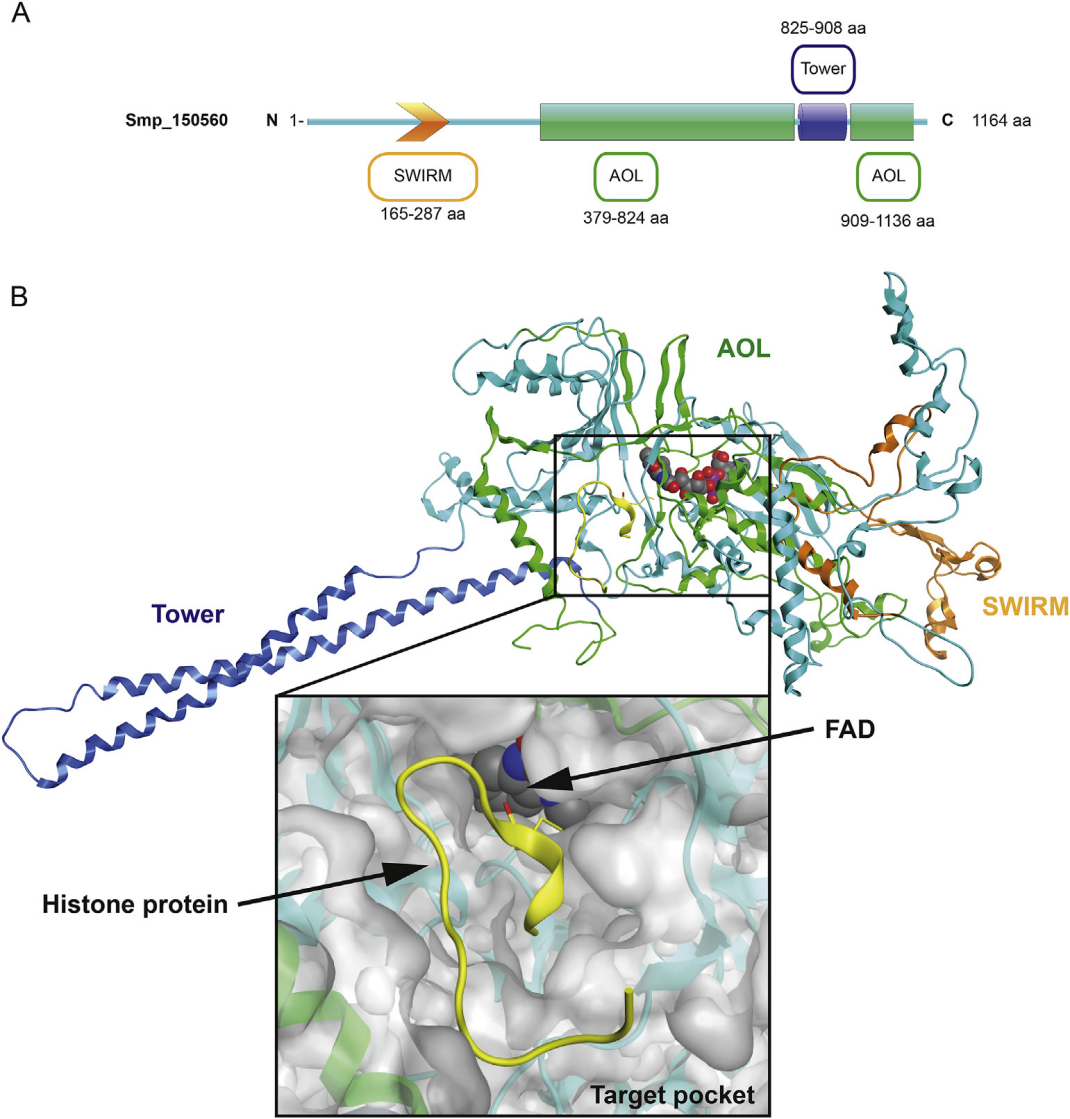
**Secondary and tertiary structural representations of Smp_150560, the closest *S. mansoni* ortholog of *Homo sapiens* Lysine Specific Demethylase (LSD) 1.(A)** Characteristic LSD1 domains found in Smp_150560 (1164 AA) include a SWIRM (orange arrow) domain, an Amine Oxidase-Like (AOL, green) domain and a tower (blue) domain. Amino acid length of each domain is provided. **(B)** Homology model of Smp_150560 in complex with the cofactor FAD and substrate histone H3 protein (20 residues fragment) produced using human LSD1 (PDB entry 2V1D) as the template. Domains are indicated and follow the colouring scheme in (A); FAD atoms are represented as spheres, histone H3 (methylated Lys is a yellow stick) is represented by yellow ribbon. Magnified target pocket (square) in SmLSD1 is represented as a surface model showing the histone (with methylated Lys) located in a channel connecting to the flavin adenine dinucleotide (FAD) cofactor (FAD shown as spheres: carbon (grey, oxygen (red), nitrogen (blue)). (For interpretation of the references to colour in this figure legend, the reader is referred to the Web version of this article.)

As there has been no comparative gene expression profiling for all SmHMTs or SmHDMs, transcript abundances of these epigenetic products were inferred from historical DNA microarray data (Fitzpatrick et al., 2009b) (Fig. 2). When compared to the only previous study examining gene expression for a single SmHMT family member (SmPRMT1, Smp_029240), our results are broadly in agreement for similarly profiled lifecycle stages (adult female and male parasites; female > male) (Mansure et al., 2005). However, our more extensive lifecycle analysis of this and all other SmHMTs (Fig. 2A) and SmHDMs (Fig. 2B) suggests that wide modulation of these family's gene expression occurs throughout schistosome development. Noticeably, all members (except the SET domain containing PKMTs Smp_144180, Smp_160700 and Smp_342100) seem to be expressed in low abundance in the daughter sporocyst lifecycle stage. Maximal abundance of any SmHMT or SmHDM was found in the sexually-mature adult lifecycle stage (5 wk old); here, the three SET domain containing PKMTs Smp_000700, Smp_078900 and Smp_210650 as well as the two JmjC containing JMJDs Smp_156290 and Smp_019170 and the AOL-containing LSD Smp_150560 are all found in higher abundance when compared to other lifecycle stages. In addition to spatial expression differences and transient variations in SmHMT and SmHDM activities, these longitudinal gene expression results are likely contributing to the previously reported developmental changes in schistosome histone methylation states and are required (together with other post-translational histone modification) for normal developmental progression in schistosomes (Roquis et al., 2015, 2018). One SmHDM, the LSD1 homolog Smp_150560 (SmLSD1, Fig. 3), was chosen as a target for further cheminformatics, functional genomics and whole organism anthelmintic studies due to the important role of human LSD1 in oncogenesis and development (Ismail et al., 2018).

### 3.3 *In silico* docking of compounds to SmLSD1

Secondary structural analysis of SmLSD1 predicted a 1164 AA protein containing a N-terminal SWIRM domain (AAs, 165–287) as well as a C-terminal amine oxidase like (AOL) domain (AAs, 379–824 and 909–1136) (Fig. 3A). Similar to HsLSD1 (Ismail et al., 2018), the AOL domain of SmLSD1 is interrupted by a Tower domain (AAs, 825–908). To progress the cheminformatics selection of putative compounds that may interfere with SmLSD1 activity, tertiary structural analysis was next conducted (Fig. 3B). However, as the structure of SmLSD1 is not currently known, we created a homology model of this schistosome HDM using chain A of HsLSD1 (PDB entry 2V1D; SmLSD1 and HsHSD1 contain 41% sequence identity over 71% of their entire length), co-crystallised with AA residues 1–16 of histone 3 (H3; position 4 is a methylated Lys) and Flavin Adenine Dinucleotide (FAD), as a template. The SmLSD1 model revealed that the SWIRM domain (orange) adopts a six-helical architecture and packs closely to the AOL domain (green). While the SWIRM domain function is still undefined, it is likely involved in DNA or protein binding (Aravind and Iyer, 2002). The AOL domain (responsible for SmLSD1 catalytic activity) is folded into two distinctive subdomains so that the cofactor binding pocket (where FAD is located, spheres) and the target-binding pocket (where methylated Lys, yellow stick, of H3 is located) are brought together. The Tower domain (blue) of SmLSD1 is likely responsible for establishing higher order protein interactions (Amente et al., 2013). The magnified surface structure of the target pocket is shown and represents the location where *in silico* binding of potential SmLSD1 inhibitors was performed.

**Fig. 4:**
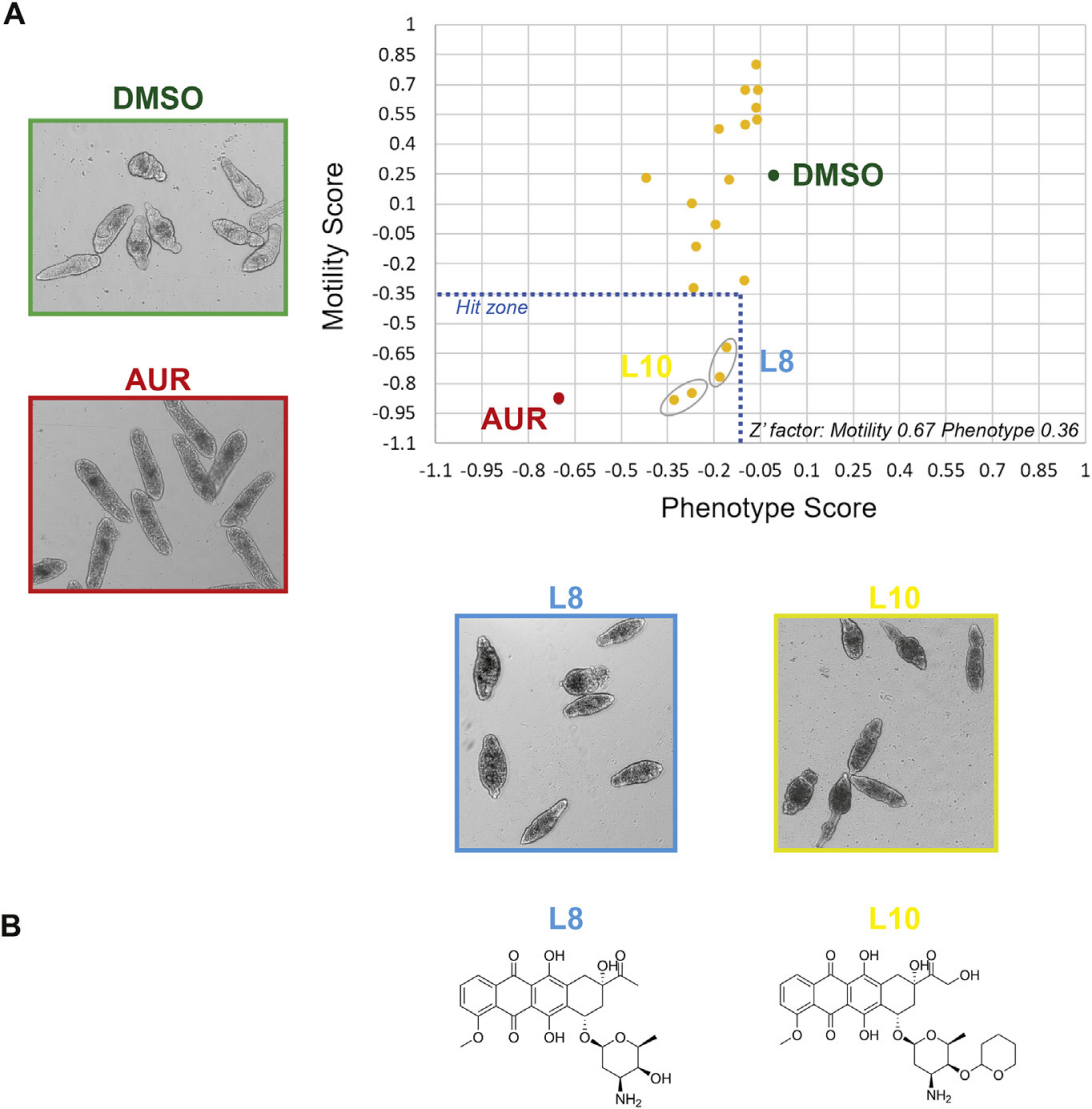
***In vitro* schistosomula screen of putative SmLSD1 inhibitors.(A)** Mechanically-transformed schistosomula (n = 120) were incubated with the putative SmLSD1 inhibitors L1 - L6 and L8 - L10 (10 μM in 0.625% DMSO; each of them in duplicate) for 72 h at 37 °C in a humidified atmosphere containing 5% CO_2_. At 72 h, the effect that each compound has on parasite phenotype and motility was assessed by the high throughput platform Roboworm. The graph shows the results of the anti-schistosomal activity of the selected compounds compared to DMSO (0.625%) and Auranofin (10 μM in 0.625% DMSO), respective negative and positive controls (average of 24 different replicate wells/control). Compounds with activity on both schistosomula phenotype and motility are shown within the ‘Hit Zone’ (delineated by the dotted lines in the graph). Z' scores (Zhang et al., 1999) for these screens are included on the graph. **(B)** Chemical structures of the two compounds (L8 and L10) showing the strongest anti-schistosomula activity.

The *in silico* molecular docking of 4532 compounds (representing a collection of both natural products and small fragment based structures (Suzuki and Miyata, 2006)) to the SmLSD1 target pocket resulted in the identification of seven compounds (L1-L7) that had a potential to interfere with the demethylase activity of this schistosome HDM (Supplementary Fig. 1, Supplementary Table 3). While we could commercially obtain compounds L1-L6, compound L7 was unavailable. Therefore, prior to conducting whole organism anthelmintic screening (Fig. 4), we identified and purchased three L7 analogues (L8, L9 and L10; Supplementary Table 3). These analogues are previously characterised anthracyclines (L8 – daunorubicin hydrochloride, L9 – doxorubicin hydrochloride, L10 – pirarubicin) with known antineoplastic activity (Minotti et al., 2004). All 10 compounds displayed a higher predicted affinity for the target pocket found in SmLSD1 compared to HsLSD1 (PDB entry 2V1D) (Supplementary Table 5).

### 3.4 *In vitro* screening of putative SmLSD1 interactors

**Fig. 5:**
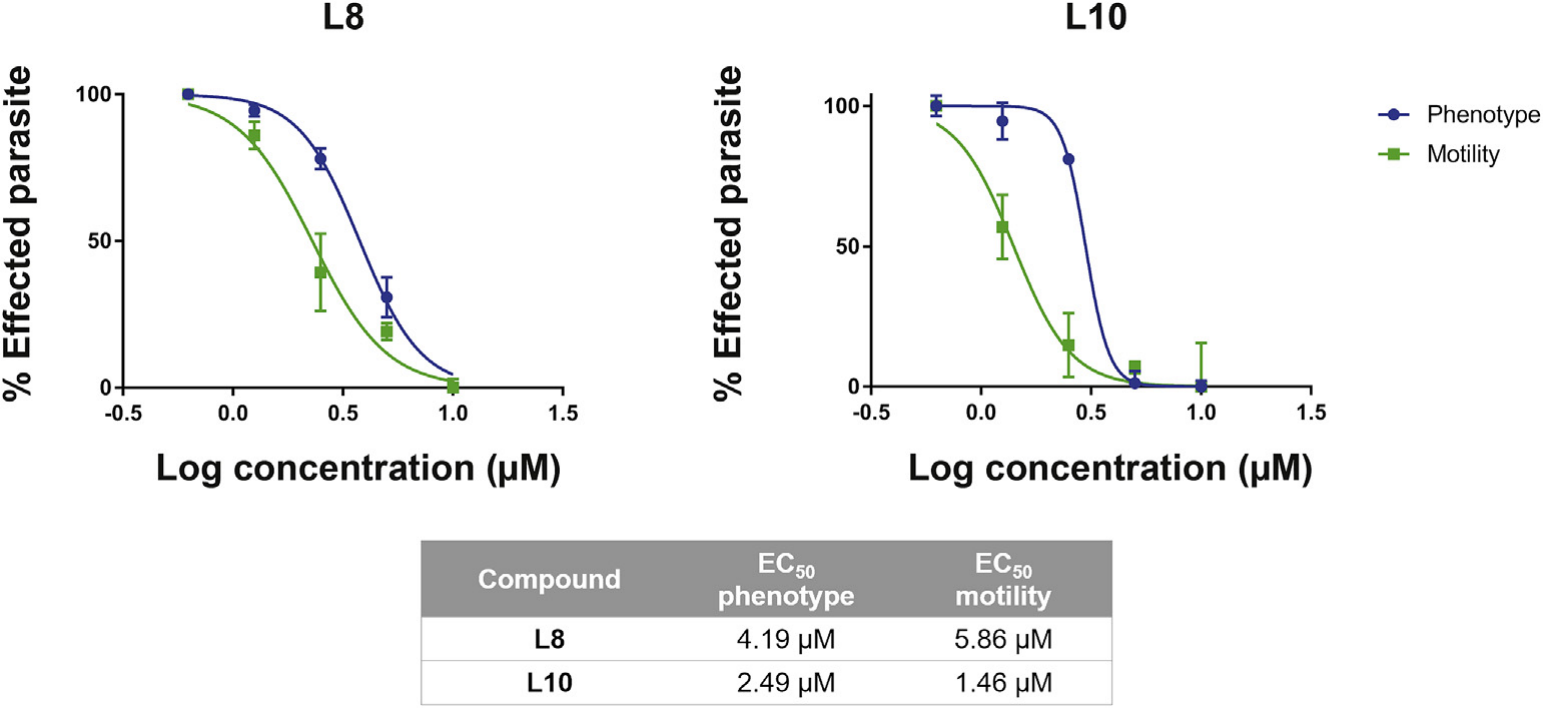
**Dose response titrations of L10 and L8 against schistosomula.** The two hit compounds were screened against mechanically-transformed schistosomula at 10 μM and lower concentrations (5 μM, 2.5 μM, 1.25 μM and 0.625 μM) as described in Fig. 4. Three independent dose response titrations were performed and each compound concentration was evaluated in duplicate. Dose response curves for *S. mansoni* schistosomula phenotype and motility were prepared using GraphPad Prism (mean ± SD of mean is indicated for each compound concentration). Estimated EC_50_s calculated from these dose response curves are summarized in the table. Z' scores (Zhang et al., 1999) for these three repeat titrations were 0.40 for motility and 0.30 for phenotype.

**Fig. 6:**
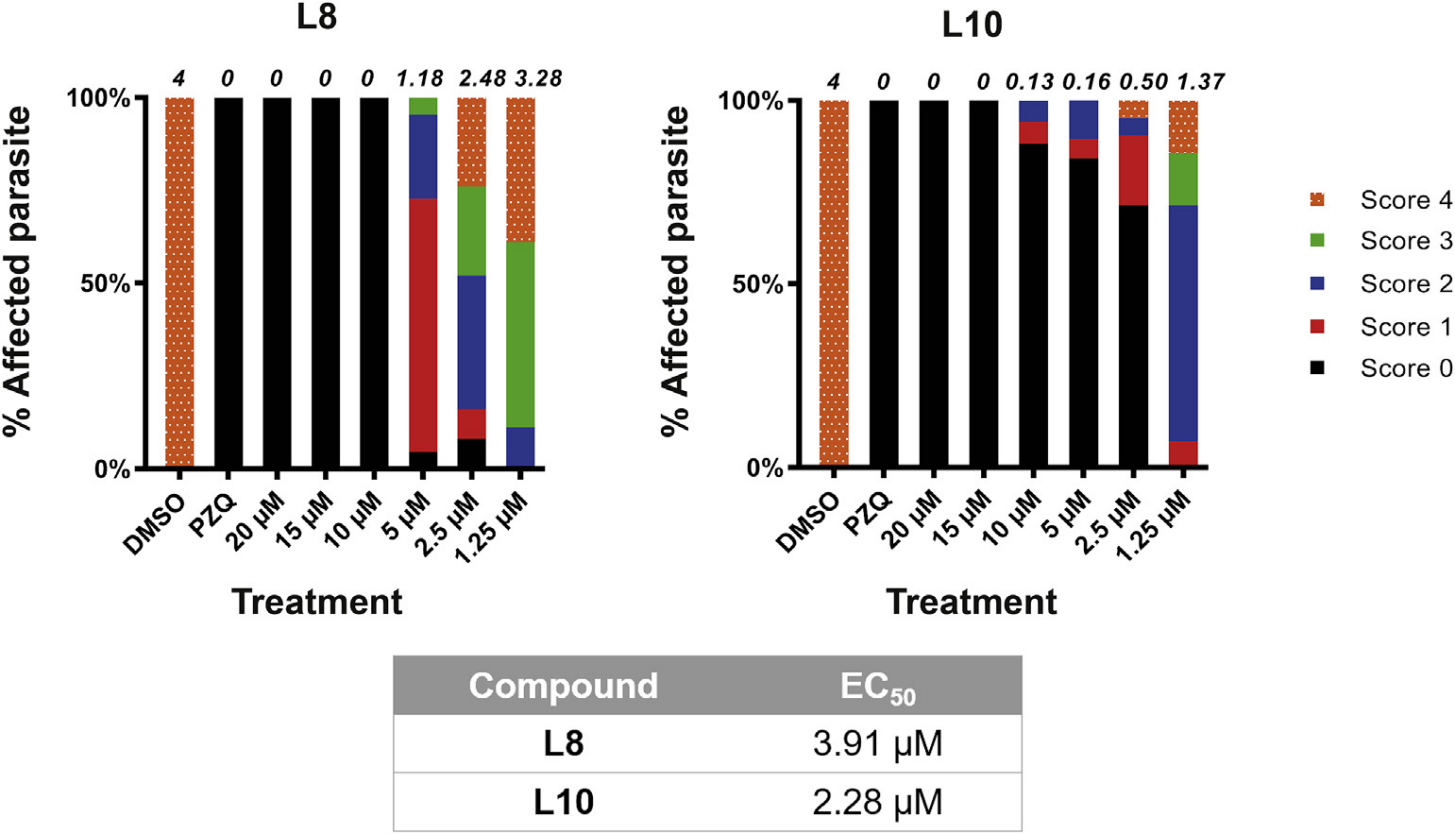
**Compounds L10 and L8 affect juvenile worm motility.** Juvenile *S. mansoni* worms (3 weeks post infection; n = 10–25) were cultured in different concentrations of L10 or L8 (20 μM, 15 μM, 10 μM, 5 μM, 2.5 μM and 1.25 μM in 1.25% DMSO) and motility scored (0 = dead, 4 = normal movement) at 72 h. Average scores/treatment are indicated on top of the stacked histograms with percentages of worms displaying the phenotype scored represented by different colour/textural combinations. Control parasites (n = 10–25) included those co-cultivated in the presence of 1.25% DMSO and PZQ (10 μM in 1.25% DMSO). Estimated EC_50_s calculated from these dose response titrations are summarized in the table. (For interpretation of the references to colour in this figure legend, the reader is referred to the Web version of this article.)

Upon screening of the nine (L1-L6; L8-10) available compounds against schistosomula on the Roboworm platform (n = 2 wells per compound/120 schistosomula per well; 10 μM single point concentration), only L8 and L10 affected both parasite phenotype and motility metrics (Fig. 4A); these two compounds were, thus, considered hits when compared to positive (auranofin (Kuntz et al., 2007)) and negative (DMSO) controls. While the central scaffold structures of L8 (daunorubicin hydrochloride) and L10 (pirarubicin) are similar (Fig. 4B), L10 is a C14-hydroxylated derivative of L8 containing a 4-tetrahydropyranyl substituent on the amino sugar in the C7 position of the central scaffold. From this primary screen, L10 appeared to be more potent than L8 against *in vitro* cultivated schistosomula (i.e. data points for L10 are closer to the origin in Fig. 4A). A subsequent dose response titration of both L10 and L8 confirmed this observation; EC_50_ values for both schistosomula motility and phenotype metrics were lower for L10 when compared to L8 (Fig. 5). This anthelmintic trend (L10 > L8) was also observed for juvenile (3 wk old) schistosomes co-cultivated with both compounds (Fig. 6). The greater anthelmintic activity of L10 over L8 may be associated with potential increased membrane permeability (L10: LogP = 2.06; L8: LogP = 1.83; Supplementary Table 3), structural differences in its sugar side chain (Supplementary Table 3 and (Denel-Bobrowska and Marczak, 2017)) or its ability to form an additional hydrogen bond with Asn653 due to the hydroxylic group in C14 within the target pocket of SmLSD1 (compare highlighted orange circles; Supplementary Fig. 3). Further medicinal chemistry investigations are necessary to disentangle these (or other) possibilities.

**Fig. 7:**
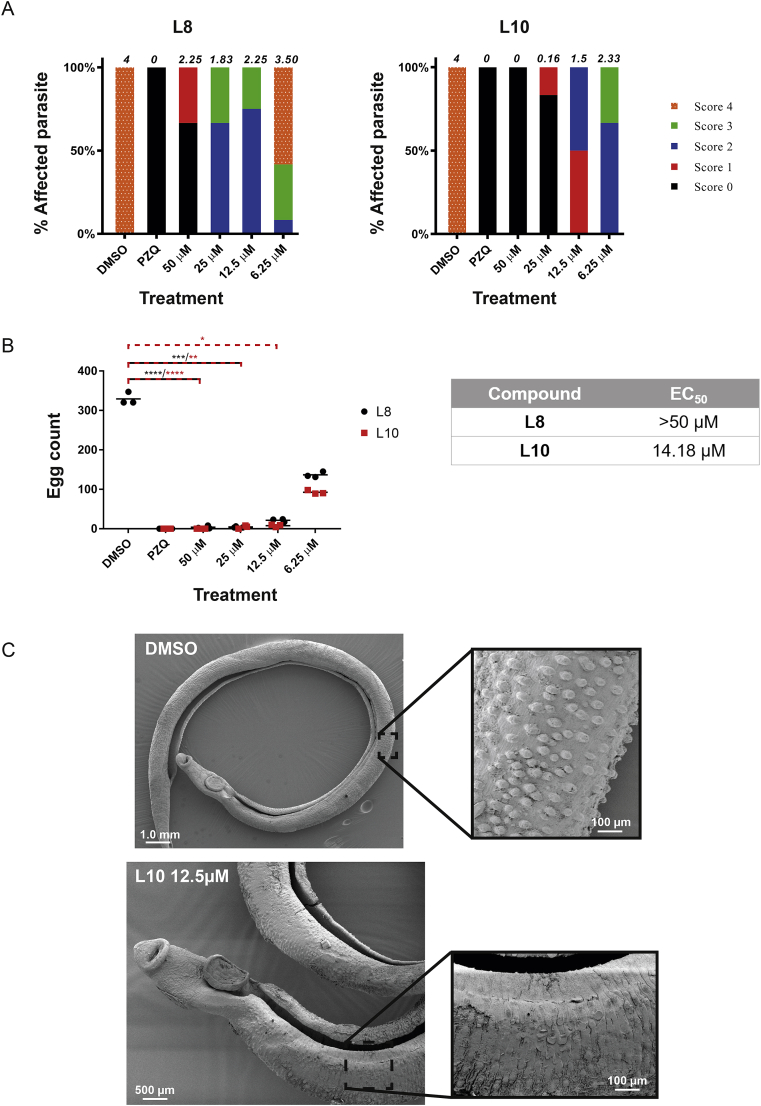
**Compounds L10 (and L8) affect adult worm motility, egg production and tegumental surface architecture.** Adult schistosome pairs (7 wk old, 3 pairs/well) were co-cultivated with L10 and L8 (two-fold titrations between 50 μM and 6.25 μM) for 72 h at 37 °C in a humidified atmosphere containing 5% CO_2_. The effect on schistosome motility **(A)** and egg production **(B)** was quantified as described in the Materials and Methods section. The stacked bars represent the percentages of worms displaying the phenotype scored and are represented by different colour/textural combinations; the average motility score (6 worms for each of the three independent experiments) is indicated above each bar. Control parasites (n = 6) included those co-cultivated in the presence of 1.25% DMSO and PZQ (10 μM in 1.25% DMSO). *, **, ***, **** represent *p* < 0.05, *p* < 0.01, *p* < 0.001, *p* < 0.0001, respectively. Estimated EC_50_s calculated from these dose response titrations are summarized in the table. **(C)** SEM analysis of adult male worms cultivated in a sub-EC_50_ concentration of L10 (12.5 μM in 1.25% DMSO) compared to DMSO controls (1.25%). (For interpretation of the references to colour in this figure legend, the reader is referred to the Web version of this article.)

We next explored the effect of both L10 and L8 on adult male and female schistosome pairs (7 wk old) (Fig. 7). Consistent with the schistosomula and juvenile worm screens, L10 displayed greater activity than L8 on adult worm motility (Fig. 7A; L10 EC_50_ = 14.18 μM, L8 EC_50_ > 50 μM). While egg production was also affected by co-cultivation with both compounds, L10 again demonstrated a more striking effect in inhibiting this critical process involved in host immunopathology and lifecycle transmission (Fig. 7B). The higher EC_50_ recorded for adult parasites (14.18 μM) compared to schistosomula (1.5 μM for motility - 2.5 μM for phenotype; Table 1), despite greater *Smlsd1* abundance in adults (Fig. 2), suggested that L10 permeability/efflux could affect the activity of the compound *in vitro* or SmLSD1 function may differ across the schistosome lifecycle. Nevertheless, incubation of *S. mansoni* adult worms with the HAT inhibitors PU139, trichostatin A, valproic acid, sirtinol, salermide and MS3 also led to worm mortality or egg production defects (Dubois et al., 2009; Lancelot et al., 2013; Carneiro et al., 2014), further supporting a critical role for the careful regulation of histone post-translational modifications in adult (and other lifecycle stages) schistosome biological processes.

**Table 1:** L8 and L10 antischistosomal activity and cytotoxicity summary.

Compound	EC_50_on schistosomula phenotupe	EC_50_on schistosomula motility	EC_50_juveniles	EC_50_ on HepG2 cells	CC_50_ on HepG2 cells	SI CC_50_/EC_50_ Schistosmula (phenotype/motility)	SI CC_50_/EC_50_ juveniles	SI CC_50_/EC_50_ Adult worms
L8	4.19μM	5.86μM	3.91μM	>50μM	17.79μM	4.25/3.04	4.55	<0.36
L10	2.49μM	1.46μM	2.28μM	14.18μM	21.05μM	8.45/14.42	9.23	1.48

As previously indicated, L10 was consistently more potent (compared to L8) in affecting schistosome phenotype or motility, regardless of lifecycle stage examined. Therefore, SEM analysis of adult male schistosomes co-cultivated in a sub-EC_50_ concentration of this compound (12.5 μM) was performed to assess if L10 also affected surface worm morphology (Fig. 7C). Here, when compared to DMSO treated control males, incubation of adult males in the presence of L10 led to the disruption and surface erosion of tegumental membranes. This type of surface damage has been seen before and is consistent with stress and an inability to maintain molecular control of this protective barrier (Edwards et al., 2015; Crusco et al., 2018). While we present molecular docking support suggesting that L10 interacts with the target pocket of SmLSD1 (Supplementary Fig. 3) and, thus, affects histone demethylase activity, it is also possible that L10 (pirarubicin) inhibits DNA and RNA synthesis, blocks transcription or produces membrane damaging free oxygen radicals as previously reported (reviewed in (McGowan et al., 2017)). Indeed, a degree of cytotoxicity was observed for L10 (and L8) using the surrogate human HepG2 cell line; however, there was a window of selectivity (i.e. selectivity index – 1.5 to 14) between this compound's anthelmintic activity and general cytotoxicity (Table 1 and Supplementary Fig. 4). Anthracycline cytotoxicity and tumour specificity is a recognized phenomenon and represents an area of growing research in developing more selective antineoplastic compounds (Edwardson et al., 2015). Nevertheless, a combination of multifactorial L10-mediated mechanisms of action (in addition to SmLSD1 inhibition) could be contributing to the anthelmintic stress response (s) observed (i.e. motility defects, surface damage and egg production inhibition); further investigations are required to tease apart L10's inhibition of SmLSD1 versus other biological processes.

### 3.5 RNAi of *Smlsd1* in adult schistosomes

**Fig. 8:**
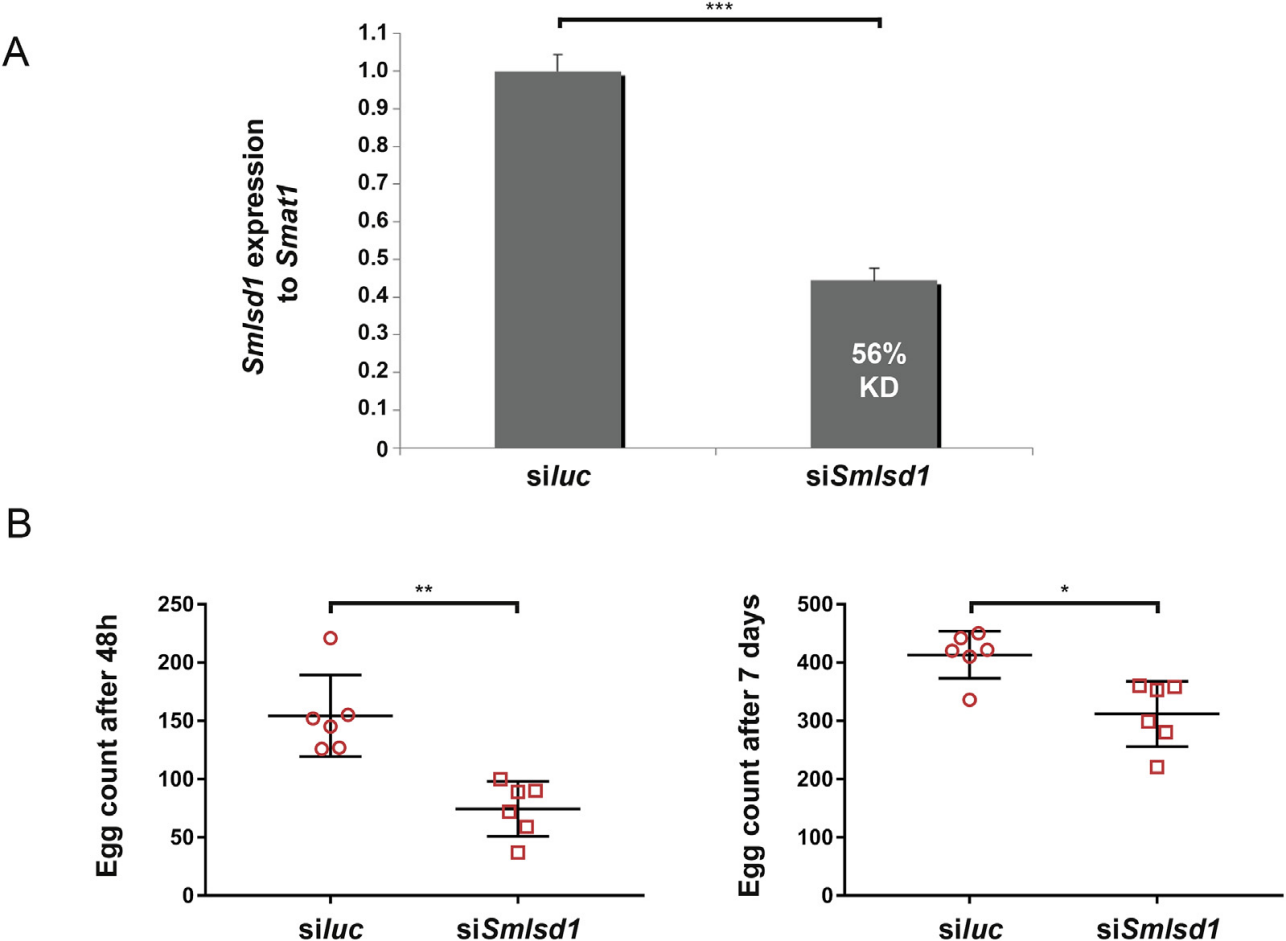
**RNAi-mediated knockdown of *Smlsd1* affects the *in vitro* production of schistosome eggs.(A)** Seven-week old adult male and female schistosomes were electroporated with 5 μg siRNA duplexes targeting luciferase (si*Luc*) or *Smlsd1* (si*Smlsd1*). Following 48 h, total RNA was harvested and subjected to qRT-PCR. Percent knockdown (KD) and statistical significance (Student's *t*-test, two tailed, unequal variance) is indicated. All siRNA and qRT-PCR DNA sequences are included in Supplementary Table 4. **(B)** Egg production at 48 and 168 h after introduction of siRNAs. Statistical significance is indicated (Student's *t*-test, two tailed, unequal variance). * represents *p* < 0.05, ** represents *p* < 0.01 and *** represents *p* < 0.001.

As a first step in progressing this objective, we undertook a functional genomics approach of SmLSD1 inhibition to see if aspects of L10-associated adult worm phenotypes could be recapitulated (Fig. 8). RNAi of adult (7 wk old) worm pairs, using *Smlsd1* siRNAs, led to a significant reduction (56%, *p* = 0.001) in transcript abundance after 48 h when compared to worm pairs treated with *siluc* siRNAs (Fig. 8A). At this time-point, egg production was also significantly inhibited (∼50% reduced) in si*Smlsd1* treated worms (Fig. 8B). While *Smlsd1* transcript reduction (56%) and egg production inhibition (50%) at 48 h is only correlated, the magnitude by which both processes were affected suggests a role for SmLSD1. After 7 days, si*Smlsd1* treated worms recovered slightly, but were still producing far less eggs than their si*luc* counterparts (Fig. 8B). While this could reflect a sustained egg production defect carrying over from 48 h, these results equally support a delay in normal egg production (compare the difference in eggs produced at the two timepoints between the two siRNA treatments). Perhaps a more significant difference in egg production (between si*Smlsd1* vs si*luc* treated worm pairs) would be observed if greater than 56% knockdown in *Smlsd1* was achieved and allow for these two scenarios to be resolved. Nevertheless, these functional genomics results, targeting *Smlsd1*, support the egg production defects mediated by L10 (Fig. 7B) and validate the molecular targeting of this *S. mansoni* HDM for follow-on investigations.

### 3.6 Conclusions

Here, we detail a strategy by which interdisciplinary approaches for pursuing drug discovery (bioinformatics, cheminformatics, whole organism screening and functional genomics) can be leveraged to identify next-generation schistosome chemotherapeutic targets. By re-classifying two opposing enzymatic families responsible for histone (and other protein) methylation and demethylation, we have identified SmLSD1 (and through homology, ShLSD1 – MS_02208 and SjLSD1 – Sjp_0082330; Supplementary Table 2) as a druggable candidate. With the further repositioning of antineoplastic compounds (L8 - daunorubicin and L10 - pirarubicin) as putative SmLSD1 (and potentially ShLSD1 and SjLSD1) inhibitors, medicinal chemistry optimisation could lead to the generation of more potent and selective anti-schistosomals that negatively affect epigenetic processes underlying schistosome development and pathogenesis.

## Conflict of interest statement

The authors certify that they have NO affiliations with or involvement in any organisation or entity with any financial interest (such as honoraria; educational grants; participation in speakers' bureaus; membership, employment, consultancies, stock ownership, or other equity interest; and expert testimony or patent-licensing arrangements), or non-financial interest (such as personal or professional relationships, affiliations, knowledge or beliefs) in the subject matter or materials discussed in this manuscript.

## Appendix A Supplementary data

Supp Fig. 1 Supp Fig. 1

Supp Fig. 2 Supp Fig. 2

Supp Fig. 3 Supp Fig. 3

Supp Fig. 4 Supp Fig. 4

Supp Table 1 Supp Table 1

Supp Table 2 Supp Table 2

Supp Table 3 Supp Table 3

Supp Table 4 Supp Table 4

Supp Table 5 Supp Table 5

Supplementary Data Legends Supplementary Data Legends

Data Profile Data Profile
