# Erratum to ‘Combining bioinformatics, cheminformatics, functional genomics and whole organism approaches for identifying epigenetic drug targets in *Schistosoma mansoni*’ [IJP Drugs Drug Resist. 8 (2018) 559–570]

**DOI:** 10.1016/j.ijpddr.2019.07.001

**Published:** 2019-07-02

**Authors:** Gilda Padalino, Salvatore Ferla, Andrea Brancale, Iain W. Chalmers, Karl F. Hoffmann

**Affiliations:** a The Institute of Biological, Environmental and Rural Sciences (IBERS), Aberystwyth University, SY23 3DA, Wales, UK; b School of Pharmacy and Pharmaceutical Sciences, Cardiff University, Cardiff, CF10 3NB, United Kingdom

The publisher regrets to inform that there was errors in the column headers of table 1 published in the original article.

The corrected table 1 is as below:

**Table 1:** L8 and L10 antischistosomal activity and cytotoxicity summary.

Compound	EC_50_ on schistosomula phenotype	EC_50_ on schistosomula motility	EC_50_ on juveniles	EC_50_ on adult worms	CC_50_ on HepG2 cells	SI CC_50_/EC_50_ schistosomula (phenotype/motility)	SI CC_50_/EC_50_ juveniles	SI CC_50_/EC_50_ adult worms
**L8**	4.19 μM	5.86 μM	3.91 μM	>50 μM	17.79 μM	4.25/3.04	4.55	<0.36
**L10**	2.49 μM	1.46 μM	2.28 μM	14.18 μM	21.05 μM	8.45/14.42	9.23	1.48

The publisher would like to apologise for any inconvenience caused.

