# Supplementary figures and images for: Combining bioinformatics, cheminformatics, functional genomics and whole organism approaches for identifying epigenetic drug targets in Schistosoma mansoni
Source: Int J Parasitol Drugs Drug Resist. 2018 Nov 13;8(3):559–70. doi: 10.1016/j.ijpddr.2018.10.005 (PMC6288008; doi:10.1016/j.ijpddr.2018.10.005)

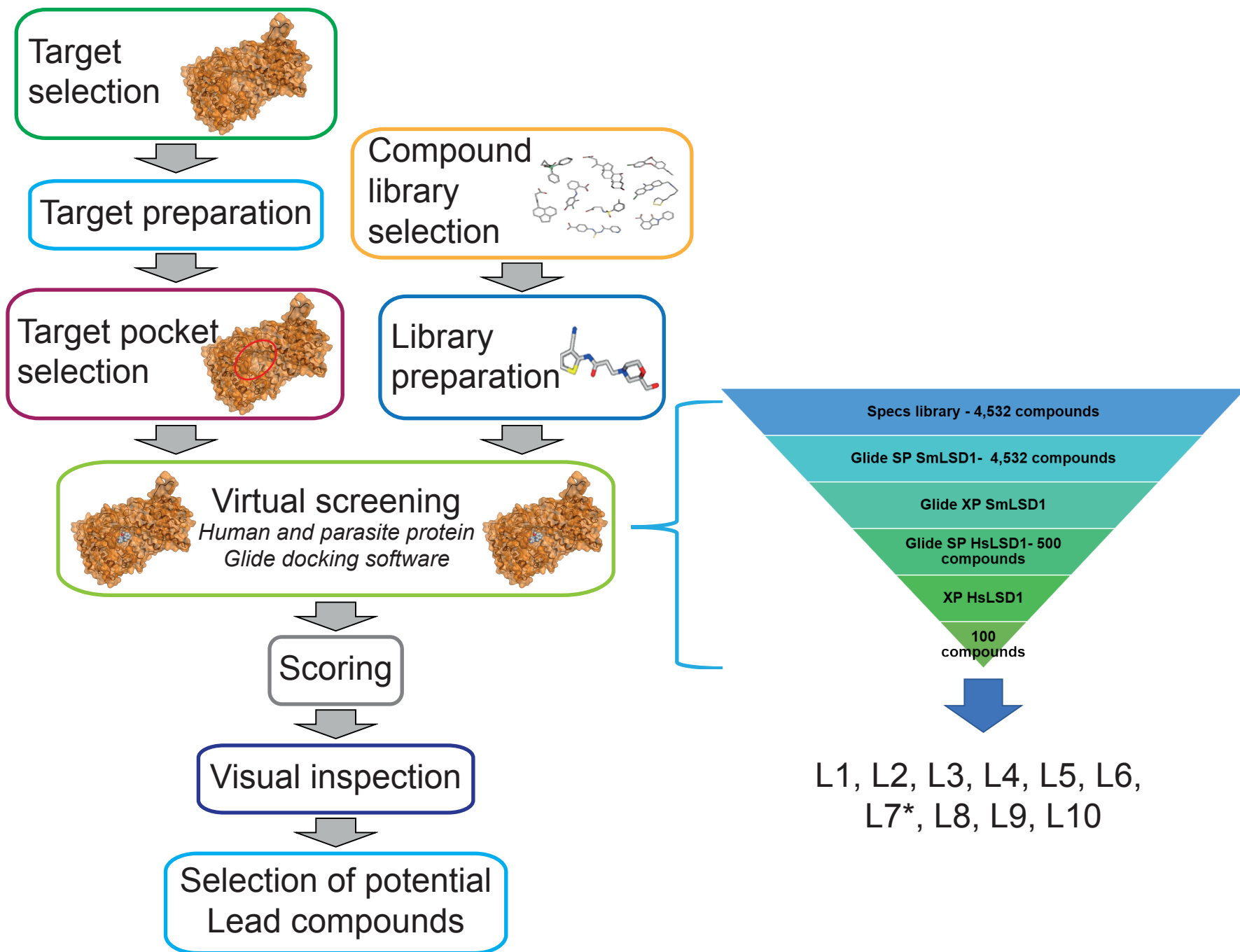

Supplement: Supp Fig. 1 [file mmc1.pdf]

A

Smp\_

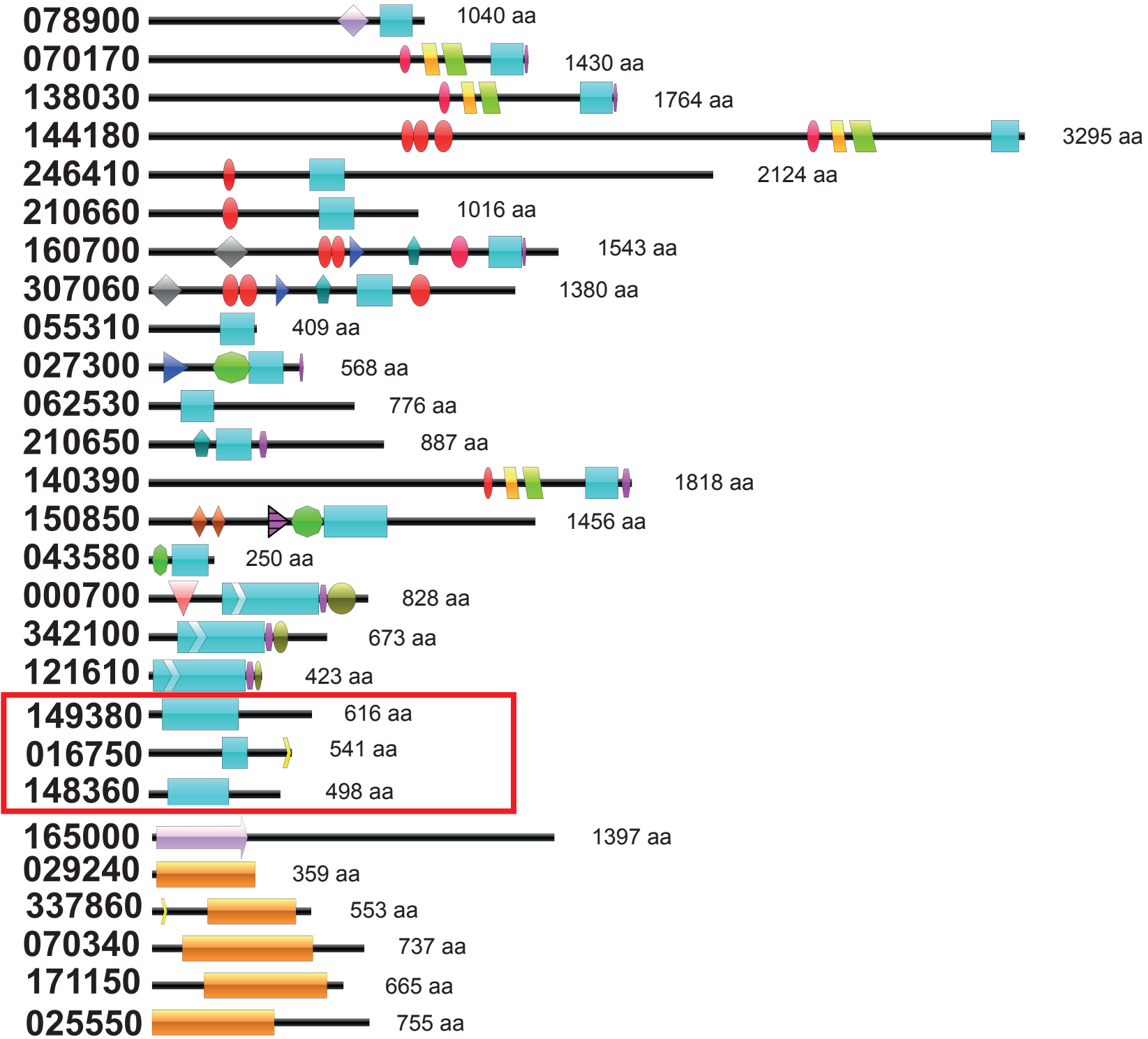H  
M  
T

B

Smp\_

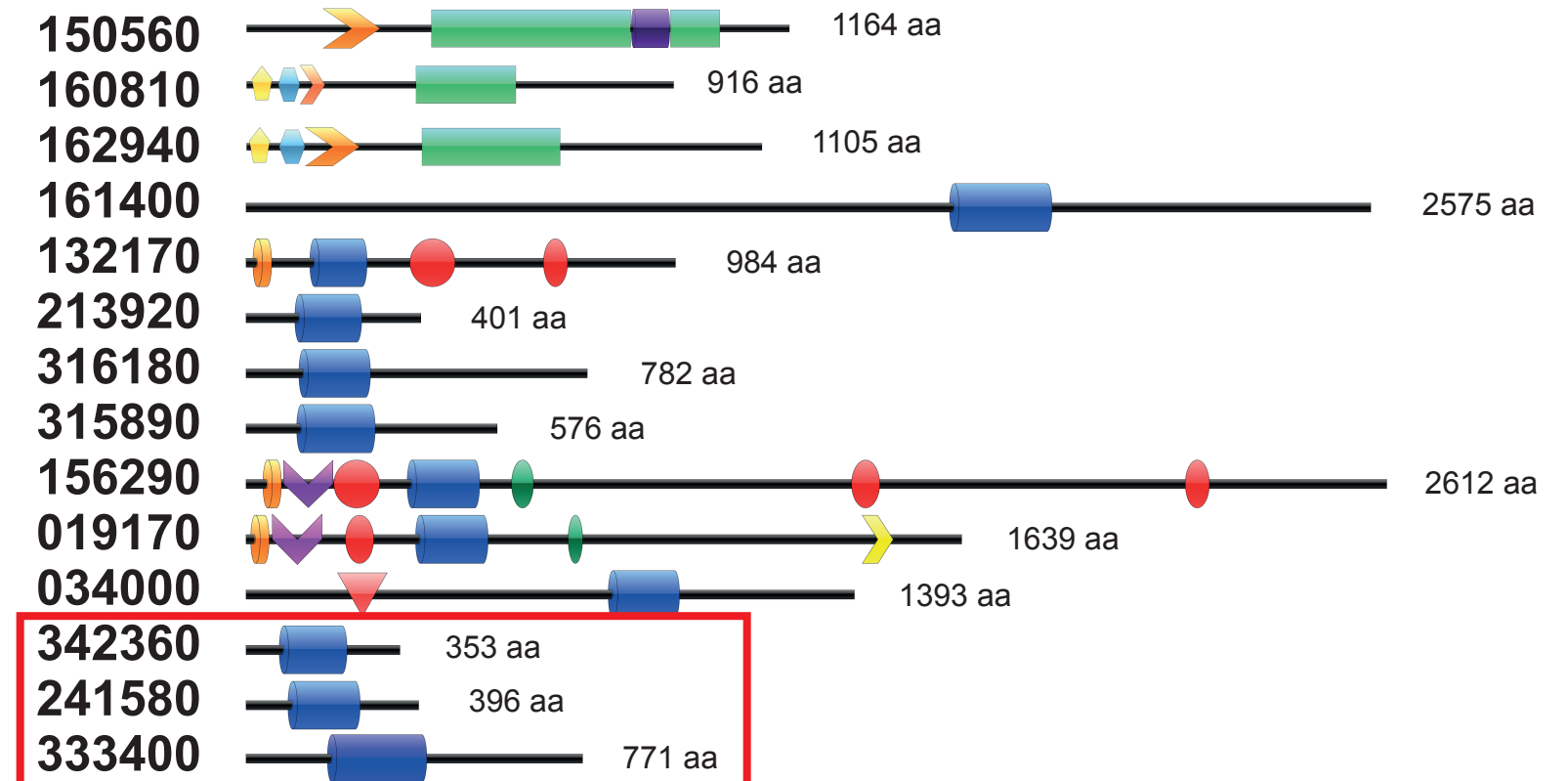H  
D  
M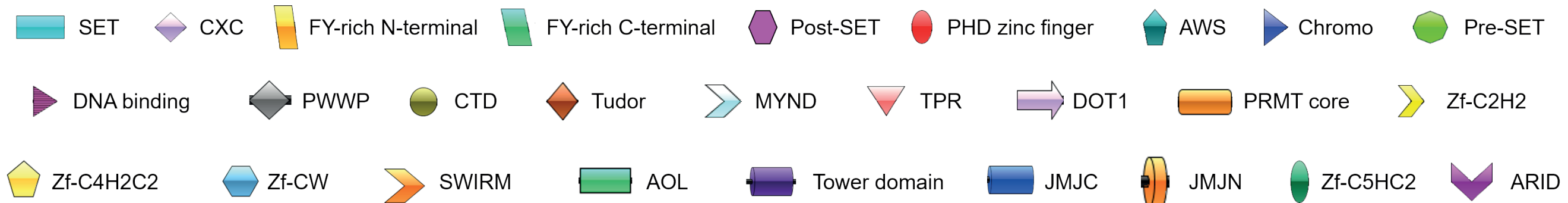

Supplement: Supp Fig. 2 [file mmc2.pdf]

## HepG2 cells

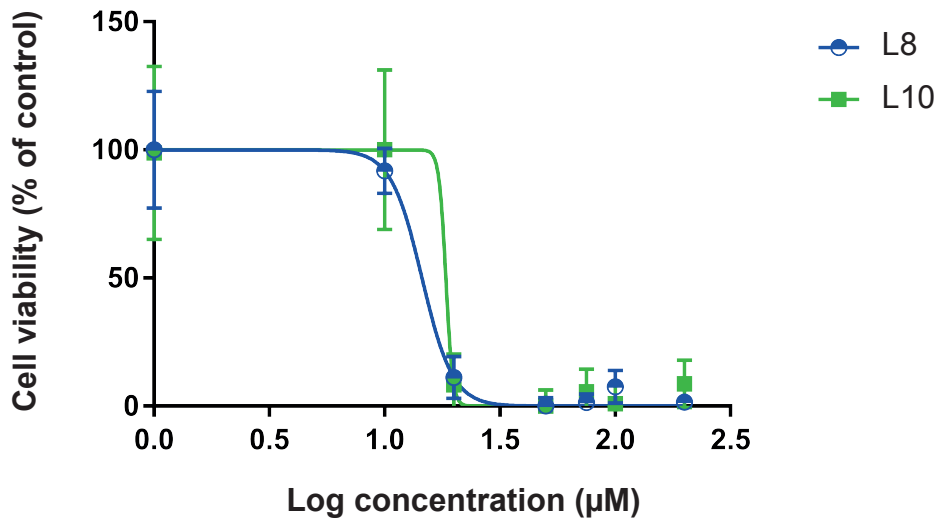

| Compound | CC <sub>50</sub> |
|----------|------------------|
| L8       | 17.79 μM         |
| L10      | 21.05 μM         |

Supplement: Supp Fig. 4 [file mmc4.pdf]
