# Supplementary material for: Combining bioinformatics, cheminformatics, functional genomics and whole organism approaches for identifying epigenetic drug targets in Schistosoma mansoni
Source: Int J Parasitol Drugs Drug Resist. 2018 Nov 13;8(3):559–70. doi: 10.1016/j.ijpddr.2018.10.005 (PMC6288008; doi:10.1016/j.ijpddr.2018.10.005)
Supplement: Supp Fig. 3 [file mmc3.pdf]

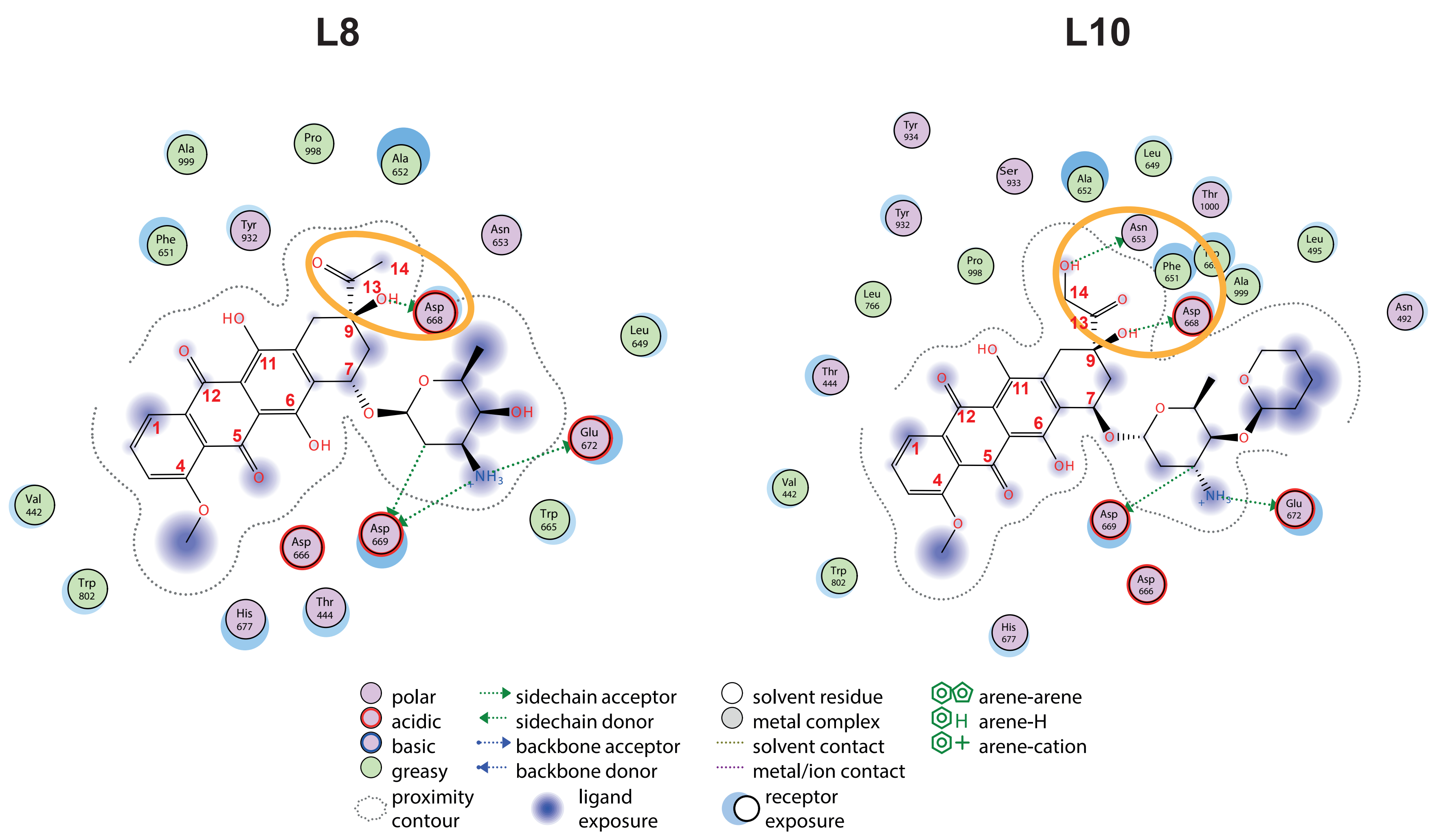
L8
L10
Ala 999
Pro 998
Ala 652
Phe 651
Tyr 932
Asn 653
Asp 668
Leu 649
Glu 672
Val 442
Trp 665
Asp 669
Asp 666
Trp 802
His 677
Thr 444
Tyr 934
Ser 933
Leu 649
Thr 1000
Leu 495
Leu 766
Ala 999
Asn 492
polar
acidic
basic
greasy
proximity contour
sidechain acceptor
sidechain donor
backbone acceptor
backbone donor
ligand exposure
solvent residue
metal complex
solvent contact
metal/ion contact
receptor exposure
arene-arene
arene-H
arene-cation
