# Supplementary material for: Combining bioinformatics, cheminformatics, functional genomics and whole organism approaches for identifying epigenetic drug targets in Schistosoma mansoni
Source: Int J Parasitol Drugs Drug Resist. 2018 Nov 13;8(3):559–70. doi: 10.1016/j.ijpddr.2018.10.005 (PMC6288008; doi:10.1016/j.ijpddr.2018.10.005)
Supplement: Supp Table 1 [file mmc5.docx]

**Supplementary Table 1. HMT and HDM domain identifiers found in protein databases.**

| HMT/HDM Subfamily | Domain Identifier | Interpro | Pfam | PROSITE | SMART |  |
| --- | --- | --- | --- | --- | --- | --- |
| PKMT | SET_dom | IPR001214 | PF00856 | PS50280 | SM00317 |  |
| DOT1L | DOT_dom | IPR025789 | PF08123 | PS51569 |  |  |
| PRMT | Arg_MeTrfase | IPR025799 |  | PS51678 |  |  |
| PRMT5 | Arg_MeTrfase_PRMT5 | IPR007857 | PF05185 |  |  |  |
| PRMT7 | MeTrfase_PRMT7 | IPR014644 |  |  |  |  |
| CARM1 | Histone-Arg_MeTrfase_N | IPR020989 | PF11531 |  |  |  |
| LSD | Amino_oxidase | IPR002937 | PF01593 |  |  |  |
| JMJD | JmjC_dom | IPR003347 | PF02373 PF13621  PF08007 | PS51184 | SM00558 | |
|  | JmjN_dom | IPR003349 | PF02375 | PS51183 | SM00545 | |
