# Supplementary material for: Combining bioinformatics, cheminformatics, functional genomics and whole organism approaches for identifying epigenetic drug targets in Schistosoma mansoni
Source: Int J Parasitol Drugs Drug Resist. 2018 Nov 13;8(3):559–70. doi: 10.1016/j.ijpddr.2018.10.005 (PMC6288008; doi:10.1016/j.ijpddr.2018.10.005)
Supplement: Supp Table 4 [file mmc8.docx]

**Supplementary Table 4. Reverse transcription quantitative real time PCR (qRT-PCR) and small interfering RNA (siRNA) oligonucleotide sequences used in this study.**

| **Common name** | **SchistoGeneDB^a^ designation** | **Oligonucleotide sequences (5´-3´)** | **Product size (bp)** |
| --- | --- | --- | --- |
| **qRT-PCR primers** | | | |
| SmLSD1 | Smp_150560 | GCCACAGGCGCTGATTATG  AGCGGCTTCACGTAGACCAC | 225 |
| SmAT1 (α-tubulin) | Smp_090120 | CTTCGAACCAGCAAATCAGA  GACACCAATCCACAAACTGG | 157 |
| **small interfering RNA (siRNA)** | | | |
| SmLSD1 | Smp_150560 | GACGUCUGGUCGAGGGUGA [dT][dT]  UCACCCUCGACCAGACGUC [dT][dT] | NA |
| Luciferase | - | CUUACGCUGAGUACUUCGA [dT][dT][dT]  UCGAAGUACUCAGCGUAAG [dT][dT][dT] | NA |

**^a^**Version 7.0
