# Supplementary material for: Combining bioinformatics, cheminformatics, functional genomics and whole organism approaches for identifying epigenetic drug targets in Schistosoma mansoni
Source: Int J Parasitol Drugs Drug Resist. 2018 Nov 13;8(3):559–70. doi: 10.1016/j.ijpddr.2018.10.005 (PMC6288008; doi:10.1016/j.ijpddr.2018.10.005)
Supplement: Supplementary Data Legends [file mmc10.docx]

**Supplementary Data**

**Supplementary Figure 1. Smp_150560 (SmLSD1) structure-based virtual screening workflow for selection of compounds to be used in whole organism schistosomula assays.** As described in Materials and Methods, two parallel strategies involving target selection/preparation and compound library selection/preparation initiate the process. Using outputs of this approach, 4,532 compounds were subjected to a virtual screening pipeline (Glide) that quantifies each compound’s ability to bind to SmLSD1’s substrate binding pocket. Using standard precision (SP) and extra precision (XP) modes, the filtering of the 4,532 compounds to 500 was achieved. The 500 putative SmLSD1 inhibitors were subjected to a virtual screening of HsLSD1 (PDB entry 2V1D) using the SP function and then the solutions were refined using the extra precision (XP) function. Ranked analyses (according to Glide XP scores) of the top 100 compounds potentially binding more selectively to SmLSD1 when compared to HsLSD1 followed and led to the selection of 7 (L1-L7) with optimal anthelmintic characteristics. Compounds 1-6 were purchased and, as L7 (*) was commercially unavailable, three structural L7 analogues (L8 - L10) were obtained. These compounds were subsequently screened for anthelmintic activity (against schistosomula).

**Supplementary Figure 2. Protein domain architecture of *S. mansoni* HMTs and HDMs.** Modular organisation of the 27 HMTs **(A)** and 14 HDMs **(B)** found in *S. mansoni* were generated from primary amino acid sequences using InterPro, Pfam, PROSITE and SMART. SET - Su(var)3-9, Enhancer-of-zeste and Trithorax domain; CXC - 65-residue cys-rich domain; FYRN - FY-rich domain N-terminal; FYRC - FY-rich domain C-terminal; Post-SET - post Su(var)3-9, Enhancer-of-zeste and Trithorax domain; PHD zinc finger - Plant HomeoDomain zinc finger; AWS - Associated With SET; Chromo - CHRromatin Organization MOdifier domain; Pre-SET - pre Su(var)3-9, Enhancer-of-zeste and Trithorax domain; PWWP - Pro-Trp-Trp-Pro domain; CTD - carboxy-terminal domain; Tudor - Tudor domain; MYND - MYeloid, Nervy and DEAF-1 domain; TPR - tetratrico peptide repeat region; DOT1 - Disruptor of the telomeric silencing 1; PRMT core - Protein Arginine Methyltransferase core domain; Zf-C4H2C2 - C4H2C2-type zinc finger; Zf-CW – CW-type zinc finger; SWIRM - SWIRM domain (named after the proteins Swi3p, Rsc8p, and Moira, in which it was first recognized); AOL - Amine Oxidase-like domain; JMJC - Jumonji C domain; C5HC2 - C5HC2-like zinc finger; ARID – AT rich interaction domain. Newly identified HMTs and HDMs are highlighted in red boxes.

**Supplementary Figure 3. L8 and L10 docking in SmLSD1’s target pocket.**

Protein-ligand interaction diagrams of L8 and L10 docked in the target pocket of SmLSD1 are shown. These 2D diagrams help in the visualization of sidechain and backbone acceptor and donor interactions. In orange circles the substituents in position 14 of the central scaffold of L8 and L10 were highlighted. The additional hydroxy group in L10 is involved in a strong hydrogen bond that can help explain the greater anthelmintic activity of L10 compared to L8.

**Supplementary Figure 4. Dose response titration of L8 and L10 on HepG2 cells.** Cells were co-cultivated with L8 and L10 according to Materials and Methods and subjected to MTT assays.

**Supplementary Table 1. HMT and HDM domain identifiers found in protein databases.** HMT and HDM domains used to help identify the 27 SmHMTs and the 14 SmHDMs.

**Supplementary Table 2. Normalised log2 expression values of *Smhmts* and *Smhdms* across *S. mansoni* lifecycle.** Worksheet one contains *Smhmt* data; worksheet two contains *Smhdm* data. Smps (*S. mansoni* genome assembly v7), DNA microarray oligonucleotide ID, 50-mer sequence and average log2 values for 14 lifecycle stages are included. *S. haematobium* and *S. japonicum* homologs of SmHMTs are contained in worksheet three; *S. haematobium* and *S. japonicum* homologs of SmHDMs are contained in worksheet four.

**Supplementary Table 3. Description of ten putative SmLSD1 inhibitors derived from from *in silico* docking experiments**. Structures, names, commercial source, SMILES and LogP are provided for each of the 10 compounds (L1-L10).

**Supplementary Table 4. Reverse transcription quantitative real time PCR (qRT-PCR) and small interfering RNA (siRNA) oligonucleotide sequences used in this study.**

**Supplementary Table 5. *In silico* docking scores of L1-L10 to the target pocket of SmLSD1 and HsLSD1.** Names, commercial compound IDs and docking scores (extra precision – XP) for all ten compounds are provided for both schistosome and human targets.
